# Regenerating human skeletal muscle forms an emerging niche in vivo to support PAX7 cells

**DOI:** 10.1038/s41556-023-01271-0

**Published:** 2023-11-02

**Authors:** Michael R. Hicks, Kholoud K. Saleh, Ben Clock, Devin E. Gibbs, Mandee Yang, Shahab Younesi, Lily Gane, Victor Gutierrez-Garcia, Haibin Xi, April D. Pyle

**Affiliations:** 1 Eli and Edythe Broad Center of Regenerative Medicine and Stem Cell Research, University of California, Los Angeles, CA USA; 2 Department of Microbiology, Immunology, and Molecular Genetics, University of California, Los Angeles, CA USA; 3 Physiology and Biophysics, University of California, Irvine, CA USA; 4 Molecular, Cellular & Integrative Physiology Program, University of California, Los Angeles, CA USA; 5 Molecular Biology Institute, University of California, Los Angeles, CA USA; 6 CIRM Bridges Program, California State University, Northridge, CA USA; 7 Jonnson Comprehensive Cancer Center, University of California, Los Angeles, CA USA

**Keywords:** Muscle stem cells, Regeneration, Musculoskeletal development

## Abstract

Skeletal muscle stem and progenitor cells including those derived from human pluripotent stem cells (hPSCs) offer an avenue towards personalized therapies and readily fuse to form human–mouse myofibres in vivo. However, skeletal muscle progenitor cells (SMPCs) inefficiently colonize chimeric stem cell niches and instead associate with human myofibres resembling foetal niches. We hypothesized competition with mouse satellite cells (SCs) prevented SMPC engraftment into the SC niche and thus generated an SC ablation mouse compatible with human engraftment. Single-nucleus RNA sequencing of SC-ablated mice identified the absence of a transient myofibre subtype during regeneration expressing *Actc1*. Similarly, ACTC1^+^ human myofibres supporting PAX7^+^ SMPCs increased in SC-ablated mice, and after re-injury we found SMPCs could now repopulate into chimeric niches. To demonstrate ACTC1^+^ myofibres are essential to supporting PAX7 SMPCs, we generated caspase-inducible ACTC1 depletion human pluripotent stem cells, and upon SMPC engraftment we found a 90% reduction in ACTC1^+^ myofibres and a 100-fold decrease in PAX7 cell numbers compared with non-induced controls. We used spatial RNA sequencing to identify key factors driving emerging human niche formation between ACTC1^+^ myofibres and PAX7^+^ SMPCs in vivo. This revealed that transient regenerating human myofibres are essential for emerging niche formation in vivo to support PAX7 SMPCs.

## Abstract (web summary)

Hicks et al. report that human regenerating myofibres expressing ACTC1 substantially contribute to supporting PAX7^+^ skeletal muscle progenitor cells.

## Main

Skeletal muscle is one of the most regenerative organs due to the presence of an endogenous muscle stem cell called the satellite cell (SC)^1,2^. SCs are supported within anatomically defined specialized compartments, termed niches, that regulate the balance of self-renewal and differentiation, similar to other highly regenerative tissues^3–5^. The SC niche is composed of dynamic ligand and receptor signalling between the myofibre sarcolemma, SC and the laminin-rich basal lamina that enables SC quiescence^6,7^. Muscle injury disrupts the SC niche inducing a regeneration cascade beginning with rapid activation of SCs to proliferative myoblasts, which turn on fusion-capable genes to form new skeletal myofibres, that will contain hundreds of myonuclei^8,9^. As new skeletal myofibres form, a subset of SCs re-enter the niche and revert from a proliferative to quiescent state, ensuring an SC pool for continual future regenerative events^10,11^.

The ability to establish new SC niches is essential for long-term cell therapies, in which transplanted muscle stem cells must balance the formation of new muscle fibres and maintain the stem cell pool^12,13^. More recently, small-molecule-mediated directed differentiation of human pluripotent stem cells (hPSCs) to skeletal muscle progenitor cells (SMPCs) has opened up the potential for personalized cell therapies and disease modelling for devastating muscle diseases from an in vitro cell source^14–17^ including in concert with gene editing^18,19^. We have demonstrated that hPSC SMPCs fuse to form hundreds of new myofibres in vivo after gene editing^20^; however, so far retention of human PAX7 cells after transplantation of hPSC SMPCs has proven challenging. Many reports show a high-magnification image of one or a few PAX7 cells following transplantation^20–22^, but to fully restore muscle function will probably require at least 8–15 engrafted PAX7 cells per 100 myofibres, similar to endogenous SC levels in adult^23,24^. Determining how transplanted SMPCs take up position in SC niches may enable support of PAX7 cells to improve long-term cell therapies for skeletal muscle diseases. The emerging SC niche (that is, the first myofibres that form upon engraftment that support PAX7 SMPCs) may also serve as an in vivo model to improve our basic understanding of human regeneration, similar to elegant studies of early development in mouse^25,26^.

Our laboratory generated a single-cell atlas of the human limb transcriptome from embryogenesis through adulthood, which determined the developmental identity of hPSC SMPCs in silico^27^. We found hPSC SMPCs most closely align with human SMPCs between weeks 7 and 12, a developmental time period that coincides with the transition from embryonic to foetal myogenesis, a period when foetal skeletal muscle is undergoing rapid growth to establish myofibres and foetal myoblasts are genetically maturing^28,29^. SMPC behaviour markedly differs from SCs including differences in mitotic activity^30^, receptor expression^31^ and extracellular matrix (ECM) composition^32^. In mouse, SMPCs migrate across and take up position under the myofibre basal lamina to establish the first niches late in foetal development^33,34^, but the mechanisms by which human SMPCs form niches and transition from progenitor to SCs to enable SC maintenance during human development or after transplantation is unknown.

In this Article, we focused on the ability to retain PAX7 cells and reside in SC niches after transplantation in vivo. We used human foetal SMPCs and adult SCs to benchmark in vivo niche occupancy and repopulate muscle after re-injury, the hallmark assay for measuring SC self-renewing potential in vivo^12^. We found that transplanted cells across all developmental states and from hPSCs had the ability to form multinucleated chimeric (mouse–human) myofibres; however, unlike adult SCs, SMPCs did not enter the chimeric mouse niche efficiently. Instead, transplanted SMPCs formed large regions of human-only myofibres and PAX7 cells formed niches with these newly formed human-only myofibres.

In the mouse embryo, it has been shown that hPSC-derived lineages are inefficient at forming niches within interspecies chimeras, but deletion of key niche genes can create a competitive niche to substantially extend hPSC donor chimerism^35^. Here we applied a similar concept by generating an immunocompromised inducible Pax7 SC ablation mouse model to study human SMPC niche formation in the absence of mouse SCs. In this model, we found an increase in human donor-derived PAX7 cells from both SCs and SMPCs but predominantly with human niches and not chimeric niches. However, upon re-injury in the Pax7-ablation model we now begin to see an increase in both human and chimeric niche occupancy demonstrating the emerging human myofibre niche supports PAX7 cells required for repopulation ability. Using Pax7-ablated and control mice as a model system to study new niche formation, we identified a transient regenerating ACTC1^+^ myofibre that is required for new niche formation during regeneration that is absent in Pax7-ablated mice. Spatial RNA profiling of engrafted stem cells and fibres identified key human skeletal muscle niche genes enabling ACTC1^+^ myofibre support of human PAX7 cells in vivo. We generated stable hPSC lines with a caspase 9-inducible kill switch to deplete ACTC1^+^ myofibres during engraftment, which we found abolished human PAX7 cells in vivo, demonstrating these ACTC1^+^ myofibres are a key component of the emerging niche needed to support human PAX7 hPSC SMPCs. In summary, this work identifies how emerging niches occur on newly regenerated myofibres in vivo and provides a new model for understanding early human stem cell niche formation using SMPCs.

## Results

### SMPCs associate with human-only myofibres in vivo

**Fig. 1:**
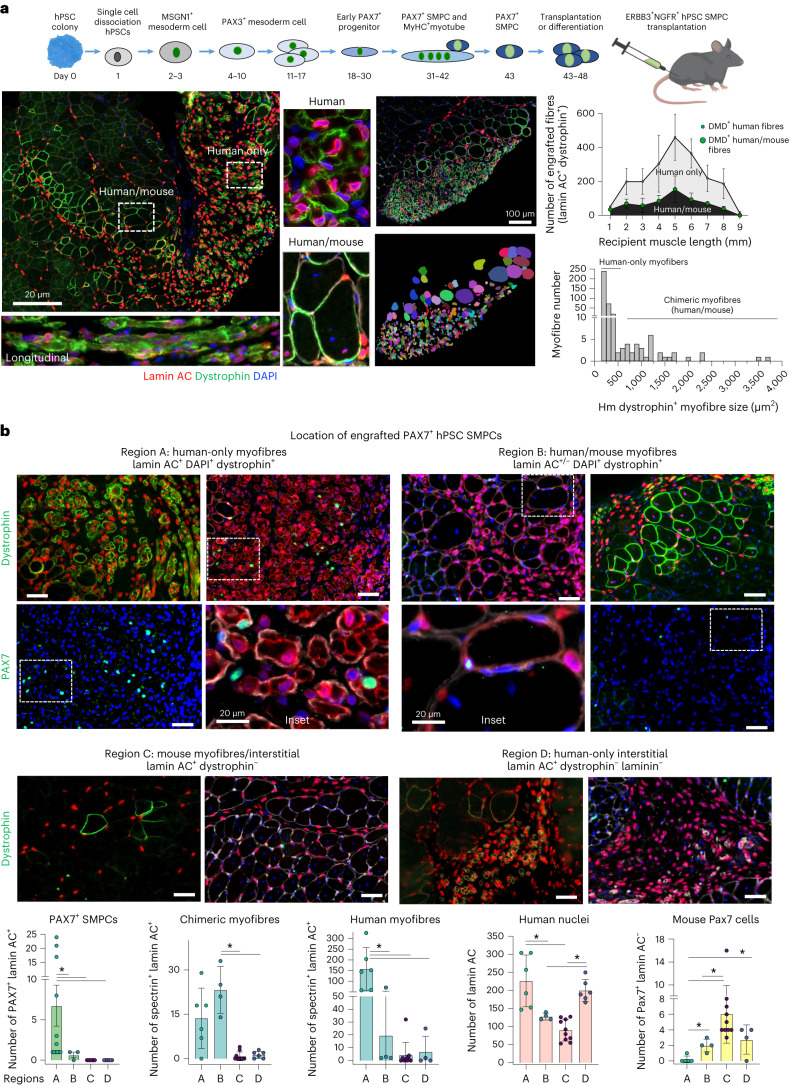
HPSC SMPC engraftment produces chimeric and human-only myofibres, and PAX7^+^ cells associate predominately near human-only myofibres. **a**, Top: schematic of hPSC directed differentiation to skeletal muscle in vitro and engraftment in vivo. Left: region of hPSC SMPCs 30 days post engraftment (1.44 mm^2^) showing insets (rotated 90°) of chimeric and human-only myofibre phenotypes. Human-specific antibodies lamin AC (red) mark human nuclei, and dystrophin (green) mark human myofibres, 4′,6-diamidino-2-phenylindole (DAPI) (nuclei, blue). Scale bar, 200 μm. Right: Imaris was used to quantify myofibre cross-sectional area as one parameter of chimeric and human-only myofibres. Scale bar, 100 μm. Graph shows mean ± standard deviation of engrafted human and human–mouse myofibres over the length of mouse TA muscle (*N* = 7). Histogram shows cross-sectional area of Imaris-identified myofibres using human dystrophin. **b**, 20× images were quantified for number of human PAX7 cells, chimeric myofibres, human-only myofibres, total human cells and mouse SCs (PAX7 green or dystrophin green depending on image; lamin AC and spectrin, red; DAPI, blue). Scale bar, 50 μm. To quantify location of PAX7 cells, numbers were then quantified using 20× images (0.15 mm^2^) were separated into four regions: (A) fields of view containing >50 human-only myofibres, (B) fields of view containing >10 chimeric muscle myofibre, (C) mouse myofibres, interstitial space and epimysium, and (D) dense numbers of human cells but few fused myofibres. Graph shows mean ± standard deviation of six to ten images from each region, *N* = 3 mice were quantified; statistics were performed using one-way ANOVA with post-hoc Tukey test, *P < 0.05. Source data

**Extended Data Fig. 1:**
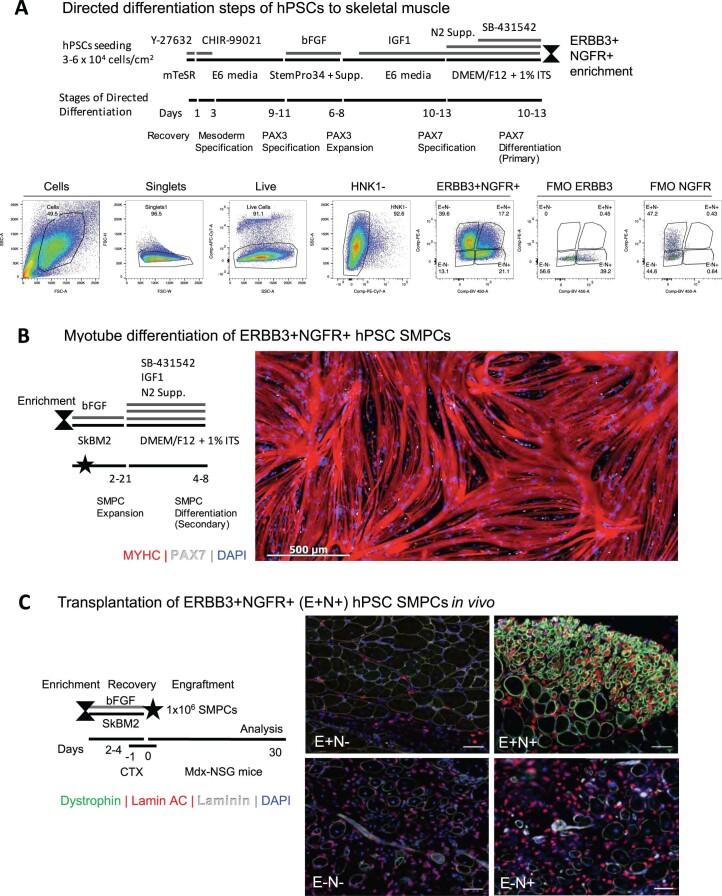
Directed Differentiation of hPSCs to Skeletal Muscle *in vitro* and *in vivo*. Related to Fig. 1a. **a**. Timeline shows stages and small molecules used for directed differentiation of hPSCs to skeletal muscle *in vitro*. Range of days for each stage indicates variance in hPSC lines and switching media conditions is based on morphology. FACS enrichment (black hourglass) shows example FACS plot of ERBB3+NGFR+ enrichment. **b**. Timeline shows expansion of ERBB3+NGFR+ hPSC SMPCs and differentiation to secondary myotubes (MYHC, red) and PAX7 cells (white). **c**. Timeline shows hPSC SMPCs expansion in SkBM2 followed by engraftment (star) into mdx-NSG mice. FACS enriched of populations ERBB3-NGFR-, ERBB3-NGFR+, ERBB3+NGFR-, and ERBB3+NGFR+ engraftment potential after 30 days and show improved engraftment by ERBB3+NGFR+ *in vivo*. ERBB3+NGFR+ show two phenotypes related to myofiber fusion showing human-only myofiber formation (top) or chimeric myofiber fusion (bottom).

**Extended Data Fig. 2:**
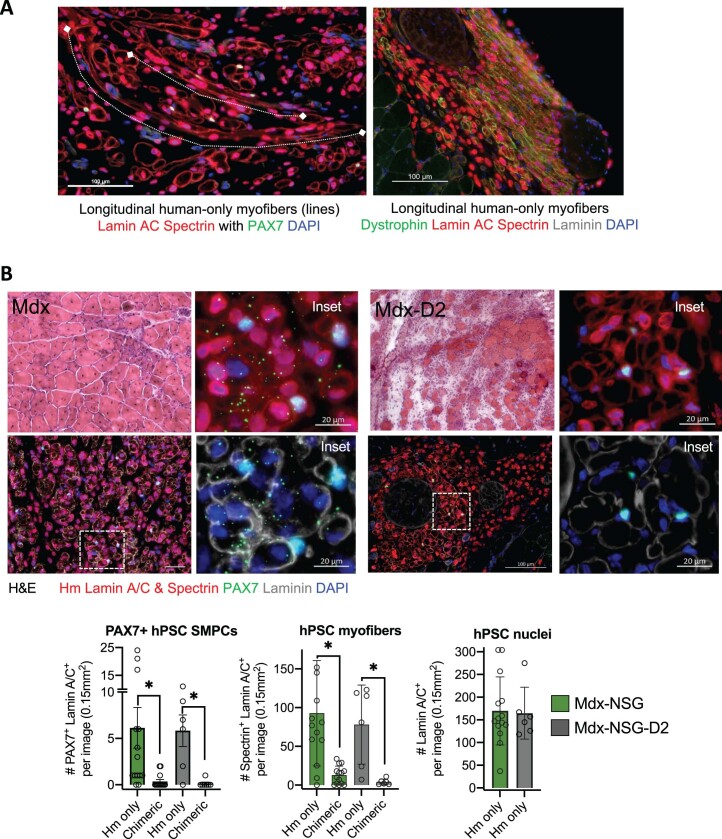
HPSC PAX7+ cells associate predominately near human-only myofibers in two dystrophic mouse models. Related to Fig. 1b. **a**. **Left**. Longitudinal sections show all nuclei (DAPI) in human-only myofibers are Lamin AC+ (red) and nearby PAX7+ hPSC SMPCs (green). Lines show beginning and end of myofiber (Spectrin, red). **Right**. Representative longitudinal image show Dystrophin+ (green) human-only myofibers and nearby neuronal bundles (laminin, grey). **b**. Hematoxylin and eosin staining of mdx-NSG and mdx-D2-NSG tissue show levels of muscle degeneration. Fluorescent images show PAX7+ cells (green) associate predominately near human-only myofibers (Spectrin+ LaminAC+, Red) in both mdx-NSG and mdx-D2-NSG mice. Inset shows zoomed in region of the engrafted hPSC skeletal muscle niche. Graphs quantify mean ± SEM 20X images (0.15mm^2^) containing PAX7 cells for number of human PAX7 cells, chimeric myofibers, human-only myofibers, and total human nuclei. A total of 15-18 images containing PAX7+ cells were quantified; statistics comparing PAX7 location performed using t-test, N=6, * = *P = 0.02* for PAX7 and * = *P = 0.0008* for human only myofibers.

We previously showed hPSC SMPCs can engraft and regenerate hundreds of dystrophin^+^ myofibres in immunocompromised mouse models of Duchenne muscular dystrophy (mdx-NSG) following cardiotoxin (CTX) injury^20^. We have continued to improve myogenic potential by supplementing survival/maturation factors during directed differentiation of hPSCs in vitro, as well as reducing culture time of ERBB3^+^NGFR^+^PAX7^+^ hPSC SMPCs before engraftment (Fig. 1a and Extended Data Fig. 1a–c). In vivo, we show that, while hPSC SMPCs fused with mouse skeletal muscle to form hundreds of multinucleated chimeric myofibres (maximum number 75–230 per cross sectional area (CSA), *N* = 7 mice), hPSC SMPCs also generated about twice as many human-only myofibres (maximum number 140–450 per CSA), which occupied large regions of engrafted skeletal muscle (Supplementary Video 1). Myofibre size as measured by Imaris software and exclusivity of human nuclei marked by human lamin AC were used to distinguish and quantify chimeric and human-only myofibres in transverse and longitudinal sections (Fig. 1a and Extended Data Fig. 2a).

An effective cell therapy must maintain the muscle stem cell pool. Yet so far retention of human PAX7 cells after transplantation of hPSC or foetal SMPCs has proven challenging. Thus, we were encouraged to find 20 or more human PAX7^+^ hPSC SMPCs within 0.15 mm^2^ (20×). A total of 127 human PAX7^+^ cells were counted and categorized on the basis of proximity to human-only myofibres, chimeric myofibres, interstitial space or regions with high numbers of human lamin AC cells but few myofibres (Fig. 1b). We found regions containing the greatest numbers of human-only myofibres also contained 98% of all engrafted PAX7 hPSC SMPCs. In contrast, regions containing only lamin AC, but few or no human-only myofibres, did not contain PAX7 cells. We also evaluated chimeric myofibre regions, and found these regions contained the other 2% of PAX7 cells (*P* < 0.05, Fig. 1b and Extended Data Fig. 2b). We also evaluated hPSC SMPC engraftment in another more severe model of muscular dystrophy, the mdx-DBA/2-NSG mice (Extended Data Fig. 2b). We similarly found that hPSC SMPCs form both human-only and chimeric myofibres, but PAX7^+^ cells predominately associated with the human-only myofibres (Fig. 1b). These data suggested that human-only myofibres were better at supporting PAX7 SMPC retention than chimeric myofibres or other regions within the skeletal muscle microenvironment.

### In vivo niches from SMPCs resemble early foetal niches

**Fig. 2:**
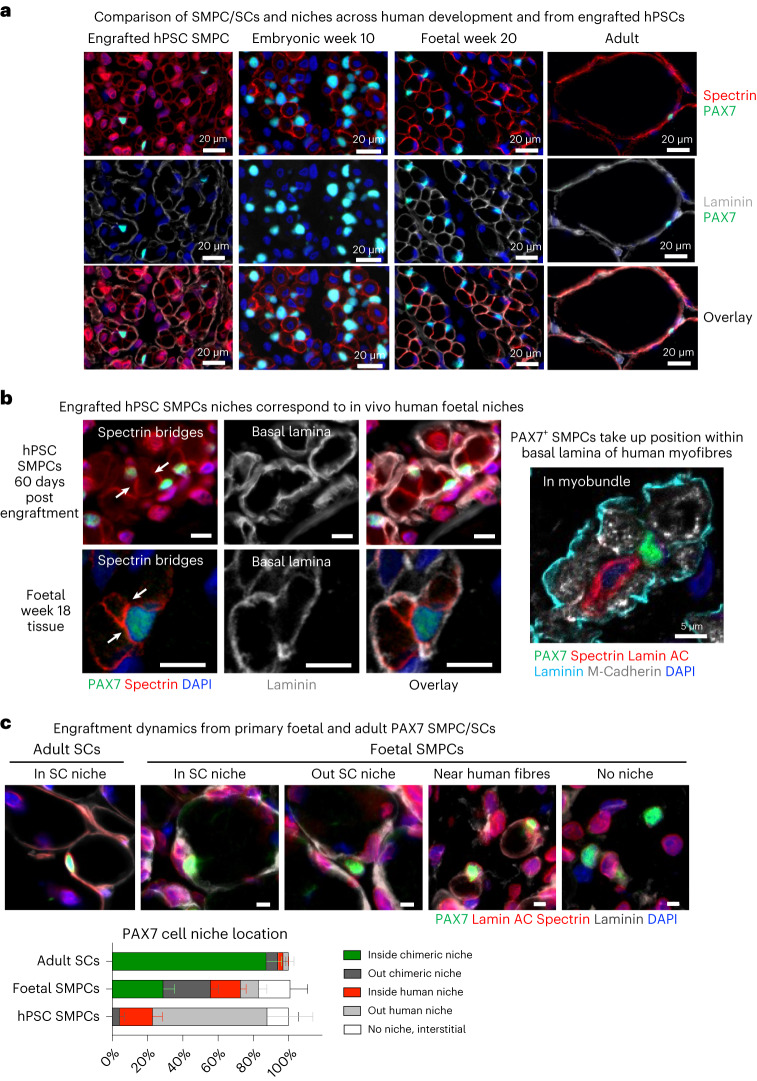
Stem and progenitor cells generate different niches, and the ability to enter the niche changes as development proceeds. **a**. Human skeletal muscle tissues show foetal and adult PAX7 + SMPC/SCs in niches compared with hPSC SMPC engraftment. Immunofluorescent images show PAX7 (green), spectrin (red), laminin (grey) and 4′,6-diamidino-2-phenylindole (DAPI) (blue). Engrafted hPSC SMPCs are also stained with lamin AC (red) to identify human cells. *N* = 3 human tissue samples. Scale bar, 20 μm. **b**, Over time, PAX7 SMPCs associate within the basal lamina niches through association with human-only myofibres, which generate myofibre bundles integrated with spectrin cross bridges (arrows). Scale bar, 10 μm (left). Phenotypically, hPSC SMPC niches resemble foetal SMPC niches in vivo with formation of spectrin cross bridges within the myofibre bundles. Scale bar, 5 μm (right). **c**, Dynamics of SC niche occupancy by PAX7^+^ SCs and SMPCs 30 days post engraftment. Immunofluorescent images show representative location of PAX7 cells in chimeric SC niches, outside of chimeric SC niches, near human myofibres, or no niche interstitial space. Shown are PAX7 (green), lamin AC and spectrin (red), laminin (grey) and DAPI (blue). Scale bar, 5 μm. Graph of percentage mean ± standard error of the mean shows that adult SCs are better able to reside in niches of chimeric myofibres, while hPSC and foetal SMPCs are less efficient. *N* = 6 adult, *N* = 9 foetal, *N* = 14 hPSC SMPC engrafted tissues. Source data

Recent work from our lab has shown that directed differentiation of hPSCs generates embryonic or foetal-like skeletal muscle in vitro^27^ and we hypothesized that engrafted hPSC SMPCs would produce similar immature skeletal muscle in vivo. To understand the developmental trajectory of transplanted PAX7^+^ hPSC SMPCs, we compared PAX7 cells and their niches with human skeletal muscle tissues from embryonic weeks 9–11, foetal weeks 16–20 and adult years 25–40 (Fig. 2a). We found embryonic and foetal myofibres were ∼30-fold smaller than adult myofibres, and this was similar to the small human-only myofibres observed after hPSC SMPC transplantation. PAX7^+^ cells in human embryonic week 10 tissues closely associated with the sarcolemma of embryonic myofibres that clustered together but lacked basal lamina expression of laminin. As myofibres matured from the embryonic to foetal stage, PAX7^+^ cells associated with the sarcolemma became encompassed by laminin, demonstrating the emergence of human niches (Fig. 2a). Here the interior sarcolemma lacked a laminin-rich basal lamina, but formed spectrin^+^ cross bridges, which are a known hallmark of myogenic fusion^36^. Similarly, several engrafted hPSC myofibres could be found in regions undergoing potential fusion in myobundles marked by spectrin that were encompassed by laminin basal lamina. Both foetal week 20 tissues and engrafted hPSCs contained PAX7^+^ SMPCs within these myobundles (Fig. 2b). These data suggested hPSC SMPCs formed niches that more closely resembled a foetal week 20 phenotype upon engraftment in vivo. This was different than adult SC niches/myofibres, which are primarily in homeostatic state where all spectrin^+^ sarcolemma was closely juxtaposed to a laminin-rich basal lamina.

### SMPCs and SCs differ in niche occupancy in vivo

We next set out to compare niche acquisition ability of human muscle stem and progenitor cells. We performed fluorescence-activated cell sorting enrichment on embryonic/foetal SMPCs (weeks 8–20, Lin-ERBB3^+^NGFR^+^) or adult SCs (years 25–60, Lin-CD82^+^NCAM^+^) dissociated from primary human tissues and immediately transplanted human cells into mdx-NSG mice. These data were used to determine whether niche acquisition resembled the endogenous niches from which the stem and progenitor cells were derived.

**Extended Data Fig. 3:**
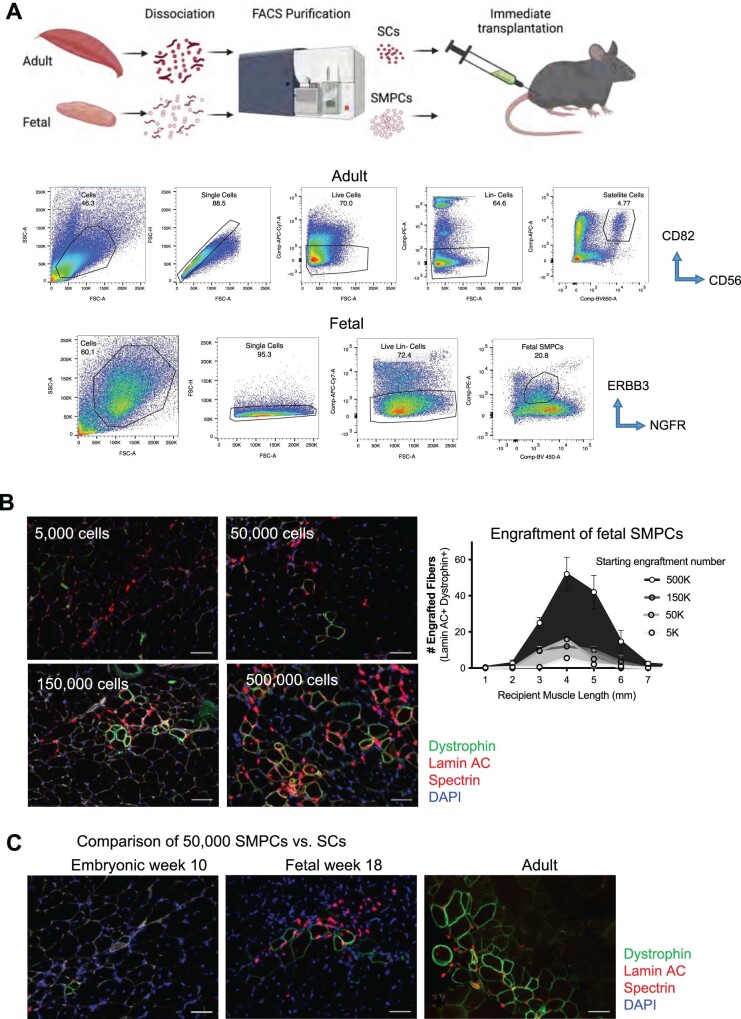
Optimization of human muscle stem and progenitor cell engraftment. Related to Fig. 2. **a**. Cartoon depicts dissociation and enrichment of primary adult stem cell (SC) and fetal progenitor cells (SMPCs) for transplantation into mdx-NSG mice. FACS plot shows enrichment for lineage negative (Lin-, CD45-CD235-PDGFRA-), SCs (CD82+CD56+), and fetal SMPCs (ERBB3+NGFR+). **b**. Comparison of dose dependent engraftment (5,000-500,000 cells) using fetal week 17 SMPCs, N = 3 human specimens in different mice. Lamin AC (red), Dystrophin (green), DAPI (blue). Graph shows mean ± SEM quantification of Lamin AC+ Dystrophin+ myofibers across the TA muscle in mdx-NSG mice. **c**. Comparison of 50,000 embryonic and fetal SMPCs and adult SCs 30 days post engraftment, show adult SCs are more able to form myofibers than fetal SMPCs at 50K engrafted cells.

We first tested engraftment efficiency across a range of foetal SMPC numbers, from 10,000 to 500,000. Foetal SMPCs (weeks 17–20) produced >100 human myofibres per cross section when 500,000 foetal SMPCs were transplanted, but at lower densities, foetal SMPCs did not restore dystrophin efficiently and only 0–1 PAX7 cells remained at day 30 (Extended Data Fig. 3). Due to embryonic tissue size (weeks 8–10) we could only obtain 50–75,000 embryonic SMPCs, and these formed myofibres poorly upon engraftment and no PAX7 cells were found, which could have been related to survival or immaturity as seen in other stem cell systems^37^. In contrast, adult SCs fused to form dystrophin^+^ myofibres and retained 5–20 PAX7^+^ SCs per cross section at 30 days in vivo with as few as 5,000 transplanted SCs (Fig. 2c and Extended Data Fig. 3). Furthermore, more than 80% of PAX7^+^ adult SCs were located in human–mouse chimeric myofibre niches 30 days post engraftment. Thus, chimeric niche acquisition is not a species compatibility issue (Fig. 2c). Foetal SMPC ability to colonize niches was intermediate between hPSC and adult SCs as we found that ∼20% foetal SMPCs were associated with small human-only myofibres and ∼30% were found inside chimeric myofibres. In contrast, 80% of PAX7^+^ hPSC SMPCs were associated within clusters of human-only myofibres and less than 5% were found within chimeric niches (Fig. 2c). These data suggest that both foetal and hPSC SMPCs can associate with human-only myofibres and ability for niche entry differs across development and adult.

### SC-ablated mice enable human PAX7 retention

**Fig. 3:**
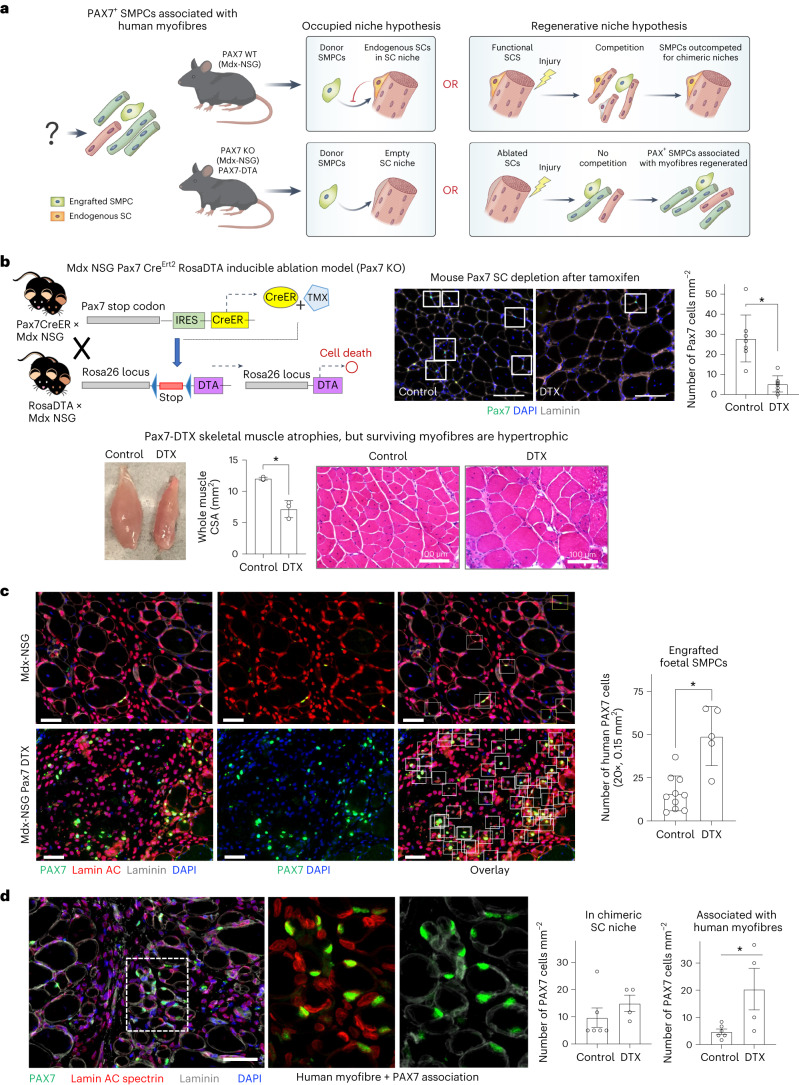
Pax7-ablation model enables evaluation of human stem cell niche formation in the absence of mouse Pax7 cell competition. **a**, Cartoon depicting hypotheses for SMPC association with human-only myofibres in the Pax7-ablation model (Pax7-ablation (DTX) mdx-NSG). Top: the occupied niche hypothesis predicts SMPCs cannot home to chimeric niches occupied by endogenous satellite cells, and the regenerative niche hypothesis predicts mouse SCs take up position in chimeric niches during regeneration. Bottom: engraftable Pax7 ablation mouse shows expected results for occupied niche and regenerative niche hypotheses. **b**, Cartoon of ablation mouse model generation and Pax7 cell numbers (white boxes) in Pax7-ablated and non-ablated control mice after 7 days of TMX treatment (resulting in Pax7 ablation or DTX), mean ± standard deviation, *N* = 5 mice, *t*-test *P = 0.0003. Tibialis anterior muscles of Pax7-ablated mice are atrophic; some individual myofibres of Pax7-ablated muscle undergo hypertrophy as measured by haematoxylin and eosin staining. **c**, Human foetal SMPCs increase PAX7^+^ numbers in Pax7-ablation mice. Representative images show co-staining of human nuclei (red), PAX7 cells (green) and 4′,6-diamidino-2-phenylindole (DAPI). White boxes indicate human PAX7 cells, and yellow boxes indicate mouse Pax7 SCs. Mean ± standard deviation of human PAX7 cells are quantified from Pax7-ablated and non-ablated mice, *N* = 5 per group, *t*-test, *P < 0.0004. **d**, Location of foetal PAX7^+^ cells are quantified 30 days post engraftment. Scale bar, 50 μm. Inset shows that human PAX7 foetal SMPCs are associated with small human-only myofibres relative to non-ablated controls, *N* = 5 per group, *t*-test, *P = 0.034. Source data

**Extended Data Fig. 4:**
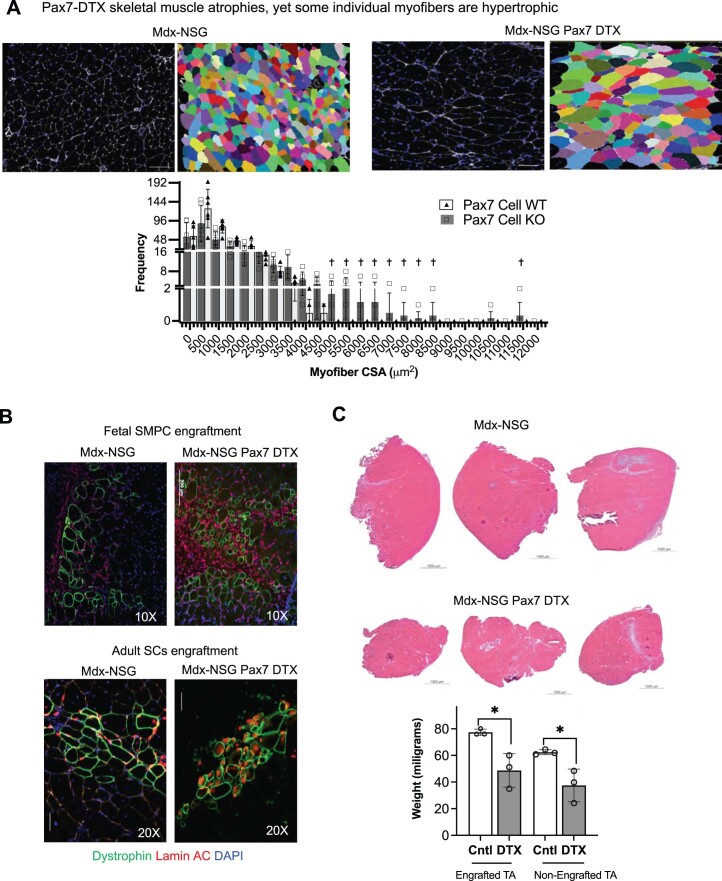
Evaluation of mouse and human myofibers after Pax7 ablation. Related to Fig. 3. **a**. Some individual myofibers of Pax7-ablated mdx-NSG muscles undergo hypertrophy as measured by laminin and Imaris software, N = 3. Histogram shows mean ± SEM frequency/mm^2^ in 500μm^2^ increments;† indicates myofiber sizes found only in Pax7-DTX mice. **b**. Representative images of engrafted human fetal SMPCs (10X) and adult SCs (20X) and into mdx-NSG and mdx-NSG Pax7-DTX mice show increased formation of small, human-only myofibers in mdx-NSG Pax7-DTX mice as measured by human Dystrophin+ Lamin A/C+, N = 4. **c**. Tibialis anterior muscles of Pax7 ablated mice are atrophic. Whole muscle cross sectional area (CSA) is decreased compared to non-ablated control (cntl) mice. Graph shows mean ± SD, * = *P<0.018*, N = 5. H&E staining of Pax7-ablated and control mice 30 days post engraftment of 1 × 10^6^ fetal SMPCs does not rescue weight loss, N = 5/mice, * = *P* = *0.025*.

As it was unclear why human PAX7^+^ hPSC and foetal SMPCs were less able to form niches with chimeric myofibres, we tested two hypotheses related to niche entry and regeneration illustrated in Fig. 3a. We generated a new inducible Pax7 ablation dystrophin model by crossing dystrophic mdx-NSG mice to Pax7-Cre/Ert2 mice and Rosa-DTX mice. When these mice are treated with tamoxifen (TMX), expression of Pax7-driven Cre recombinase removes LoxP stop codons to induce expression of diphtheria toxin (DTX) resulting in SC death^38,39^ (Fig. 3b). Thus, in this model, regeneration and niche formation would preferentially occur from transplanted human cells as competition with mouse SCs was removed. Pax7-DTX mice were evaluated for SC ablation following 7 days of TMX-infused chow, which we found was sufficient to ablate greater than 80% of endogenous Pax7 SCs relative to non-ablated controls (Fig. 3b). Following CTX- or BaCl_2_-induced injury of tibialis anterior muscles, Pax7 ablation resulted in decreased weight and cross-sectional area, demonstrating that Pax7 cells are required for muscle repair, but did not result in complete loss of all myofibres (37 mg versus 62 mg, *P* < 0.05) (Fig. 3b). We noted a subset of myofibres underwent hypertrophy after Pax7 ablation, growing up to four-fold larger than non-ablated controls, demonstrating myofibres are phenotypically influenced by loss of Pax7 SCs and their niches (*P* < 0.05, Fig. 3b and Extended Data Fig. 4a).

We next tested the ability of human foetal SMPCs to engraft and reside in the niche of Pax7-ablated mdx-NSG mice. We found a two-fold increase in PAX7^+^ foetal SMPCs 30 days post engraftment (*P* < 0.05, Fig. 3c). We evaluated the location of foetal PAX7^+^ SMPCs and surprisingly found these were less in mouse SC niches, and more associated with small human fibres upon mouse SC ablation (*P* < 0.05, Fig. 3d). While in some cases engrafted foetal SMPCs fused with mouse to form chimeric myofibres in mdx-NSG Pax7-ablated mice, we found foetal SMPCs predominately formed small human-only myofibres. Interestingly, adult SC engraftment in Pax7-DTX mice also followed a foetal trajectory where adult SCs were more likely to be found near small human-only myofibres (Fig. 5a,b and Extended Data Figs. 4b and 6c). Together, these data point to the idea that, in the absence of mouse Pax7 SCs, human-only myofibres are left in a muscle microenvironment that promotes a developmental or regenerative-like paradigm that enables the support of engrafted PAX7 SMPCs.

### Myofibres that support SC niches are lost upon SC ablation

**Fig. 4:**
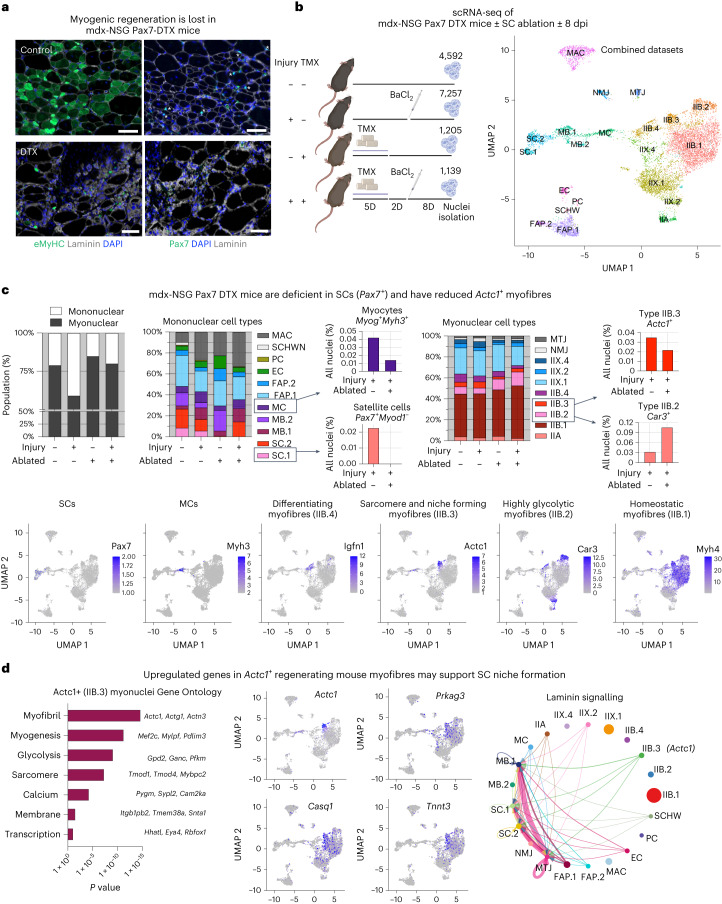
Lack of regeneration in Pax7-ablated mice is associated with decreased *Actc1*^*+*^ myofibres. **a**, Regenerative ability is lost in Pax7-ablated mdx-NSG mice 8 days post injury (dpi) as shown by lack of eMyHC (left, green) and Pax7 (right, green). Inset shows mouse Pax7 SCs (asterisk) associate with small myofibres during regeneration. *N* = 3 mice. Scale bar, 50 μm. **b**, Schematic of experimental groups show timing in days (D) of TMX treatment resulting in Pax7 ablation (DTX) and injury (BaCl_2_) of Pax7-ablated mdx-NSG mice used for snRNA-seq. UMAP of combined samples identifies 21 cell populations; abbreviations are defined in Supplementary Table 1 (*N* = 4). **c**, Left: graph shows percentage of all myonuclear (grey) and mononuclear (white) populations in response to injury or Pax7 ablation. Left centre: graphs show percentage of mononuclear cell types; boxes show enlarged view of satellite cells (SC.1) and myocytes (MC). Right: graphs show percentage of myonuclear populations; boxes show enlarged view of *Car3*^*+*^*Myh4*^*+*^ myonuclei (IIB.2) and *Actc1*^*+*^*Myh4*^*+*^ myonuclei (IIB.3). Bottom: UMAPs show spatial relationships and key genes of satellite cell, myocyte and myofibre populations during muscle regeneration. **d**, Gene Ontology of upregulated genes in *Actc1*^*+*^ myonuclei (IIB.3) is shown (*q* < 0.05). UMAPs of key upregulated genes in IIB.3 myonuclei are shown. CellChat analysis shows laminin signalling pathway network between populations with specific interaction between IIB.3 myofibres and SC.1, SC.2 and MB.1. Source data

While it is well known that Pax7 cells are essential for myofibre formation^39–41^, it is less clear how the SC niche and regeneration are affected by the absence of Pax7 cells. Therefore, we set out to identify which genes or populations were missing in the myofibres of Pax7-ablated mice. Mouse SC niches are re-established in parallel with new myofibre formation 7–10 days post-injury (dpi)^10,42^. Thus we selected this timepoint post injury to study niche formation, which we confirmed by embryonic myosin heavy chain (eMyHC) expression and increased mouse Pax7 SCs in mdx-NSG mice 8 dpi, and these cell types were both lost in mdx-NSG Pax7-DTX mice (Fig. 4a). We performed single-nucleus RNA sequencing (snRNA-seq) on Pax7-ablated muscle compared with non-ablated and regenerating muscle 8 dpi, and used recently published datasets as a starting point to identify new and known populations in our ablation models^43–45^. In total 14,300 nuclei were sequenced, enabling identification of several population shifts between regenerating, non-ablated and Pax7-ablated myofibres (Fig. 4b, deposited as GSE241368).

Seurat analysis identified a total of 21 populations including 10 myonuclear and 11 mononuclear populations (Supplementary Table 1). We found a two-fold influx in mononucleated cells in regenerating mdx-NSG mice 8 dpi, which included immune, stromal and myogenic populations (Fig. 4c). In contrast, Pax7 ablation had a blunted influx of mononucleated cells 8 dpi. Macrophages and *Itga7*^*+*^*Vim*^*+*^ myoblasts (MB.2, described by ref. ^46^) were the only mononuclear populations to increase in Pax7-ablated mice. Seurat analysis identified a *Pax7*^*+*^*Myod1*^−^ SC population (SC.1) which as expected was eliminated in Pax7 ablated muscle (0.1% ablated versus 5.6% non-ablated). In non-ablated muscle, *Myod1*^+^*Ki67*^*+*^ myoblasts (MB.1, 0.6% versus 4.5%) and *Myog*^*+*^*Myh3*^*+*^ myocytes (MC, 1.3% versus 4.2%) increased 8 dpi compared with no injury, respectively, but upon Pax7 ablation the regenerative response of myoblasts (0.7% versus 2.6%) and myocytes (0.6% versus 1.4%) was diminished 8 dpi, validating that regeneration was absent following Pax7 ablation (Fig. 4c).

We also found several changes in myonuclear populations. As sequencing was performed using tibialis anterior, the majority of myonuclei were fast-twitch glycolytic type IIB myofibres (*Myh4*^+^, 59–70%), while a minority of myofibres were type IIX (*Myh1*, 24–33%) or type IIA (*Myh2*, 3–4%) (Supplementary Table 1). Type IIB myofibres were subdivided into four populations identified as follows: IIB.1, a homeostatic *Myh4* subcluster; IIB.2, marked by the high-glycolytic-capacity genes (*Car3*, *Pkbfb3* and *Amy1*); IIB.3, marked by sarcomere assembly genes (*Actc1*, *Tmod1* and *Myhbp3*); and IIB.4, which expressed mitotic inhibition (*Trp63* and *Gadd45a*) and myogenic growth genes (*Fst1* and *Igfn1)*. Upon Pax7 ablation, *Car3*^+^ IIB.2 myonuclei increased by 2.4-fold relative to non-ablated controls, suggesting these are longer lasting and less regenerative myofibres (Fig. 4c). In non-ablated mice, upon injury the percentage of all type IIB myofibre populations decreased, except for *Actc1*^+^ IIB.3 myofibres, which increased at 8 dpi and was two-fold greater than Pax7-ablated muscle, suggesting *Actc1* marked a regenerative myonuclear population. Uniform Manifold Approximation and Projection (UMAP) reduction of myogenic regenerative response highlights that *Actc1*^*+*^ myofibres are intermediary between *Myog*^*+*^*Myh3*^*+*^ myocytes and more mature *Myh4*^*+*^ myofibres (Fig. 4c).

**Extended Data Fig. 5:**
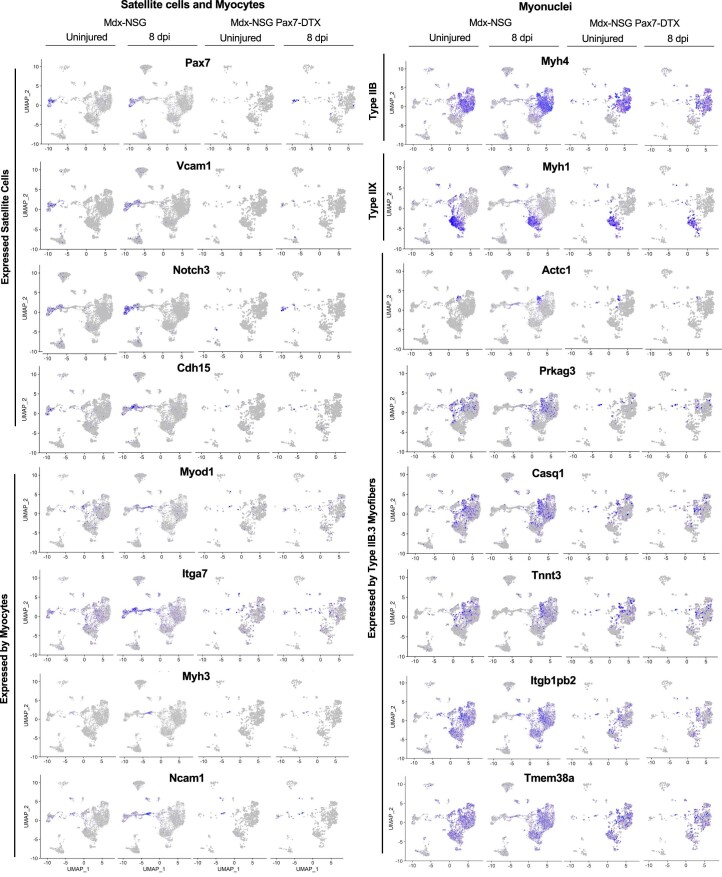
UMAPs of key myogenic genes in mouse Pax7 ablation and single nucleus RNA sequencing. Related to Fig. 4. Shown are representative individual UMAPs from mdx-NSG mice including non-injured, 8 days post injury (dpi), non-injured Pax7-ablated, and Pax7-ablated 8dpi. Notable gene expression on populations include: **Left**. satellite cells (*Pax7, Vcam1, Notch3, Cdh15*), myocytes (*Myod1, Itga7, Myh3, Ncam1*), and **Right**. Type IIB myofibers (*Myh4*), Type IIX myofibers (*Myh1*), and Type IIB.3 myofibers (*Actc1, Prkag3, Casq1, Itgb1bp2, Tmem38a*).

**Extended Data Fig. 6:**
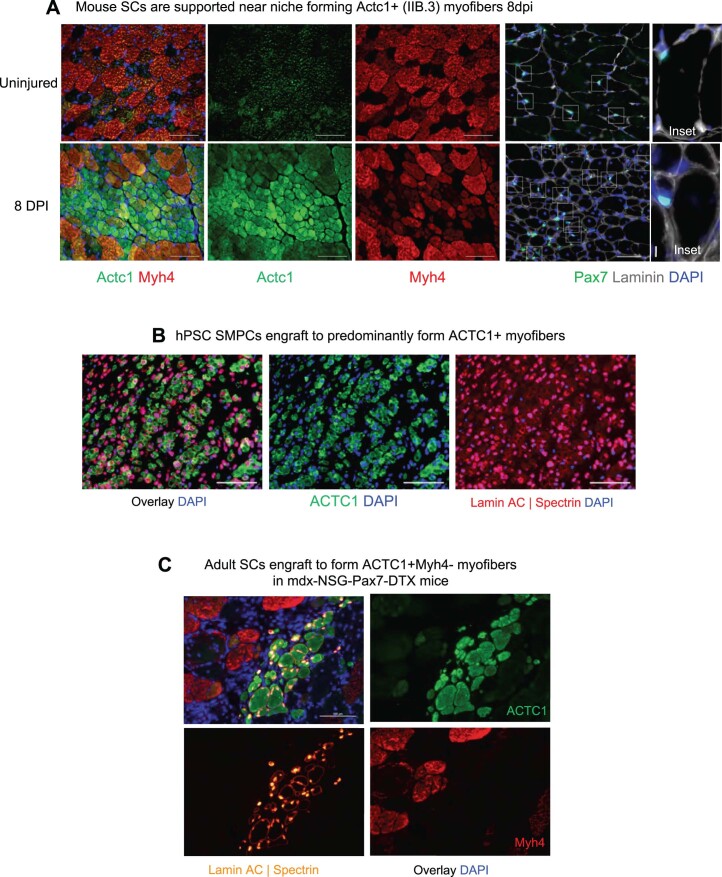
Actc1 is increased on regenerating mouse myofibers and marks ACTC1 human-only myofibers in Pax7-DTX mdx-NSG mice. Related to Fig. 5a. **a**. Actc1+ mouse myofibers (green) are markedly increased 8 days post injury (dpi). Actc1 is expressed by Type IIB mouse myofibers as shown by Myh4 (red) co-staining. Mouse Pax7 SC numbers (boxes) increase near regenerative Actc1+ myofibers. Inset highlights Pax7 cells in niches of non-regenerating and small regenerating mouse myofibers. **b**. hPSC SMPCs engraft to predominantly form ACTC1+ myofibers. LaminAC/Spectrin (red), ACTC1 (green), DAPI (blue). **c**. Engrafted human adult SCs form ACTC1+ myofibers (green) in Pax7 DTX mice but lack expression of Myh4 (red) suggesting these are human-only myofibers, 30 days post engraftment.

Since *Actc1* was the highest-enriched gene in IIB.3 myonuclei, we confirmed with immunostaining that Actc1 protein expression overlaps with timing of SC niche establishment during mouse muscle regeneration (Extended Data Fig. 6a). In contrast, while embryonic myosin protein is co-expressed on Actc1^+^ myofibres, the *Myh3* RNA expression is gone at 8 dpi. Thus, *Actc1* expression may be longer lasting than *Myh3* and may mark a myofibre important for supporting new SC niche formation. Our snRNA-seq analysis identified 134 upregulated genes expressed by Actc1^+^ (IIB.3) myofibres, which included genes important for sarcomere formation, nucleotide and lipid metabolism, mitochondrial energetics and calcium regulation (for example, *Tnnt3*, *Eya4*, *Prkag3*, *Ampd1* and *Casq1*). These myofibres also expressed membrane-associated gene signatures related to Notch, Integrin and Jak/Stat signalling (Fig. 4d, Extended Data Fig. 5 and Supplementary Table 2). We used CellChat analysis^47^ to analyse ligand–receptor niche interactions, which identified increased laminin signalling between Actc1^+^ myofibres and mouse Pax7^+^ SCs compared with other myofibre populations (Fig. 4d).

### ACTC1^+^ myofibres enable repopulation in SC-ablated mice

**Fig. 5:**
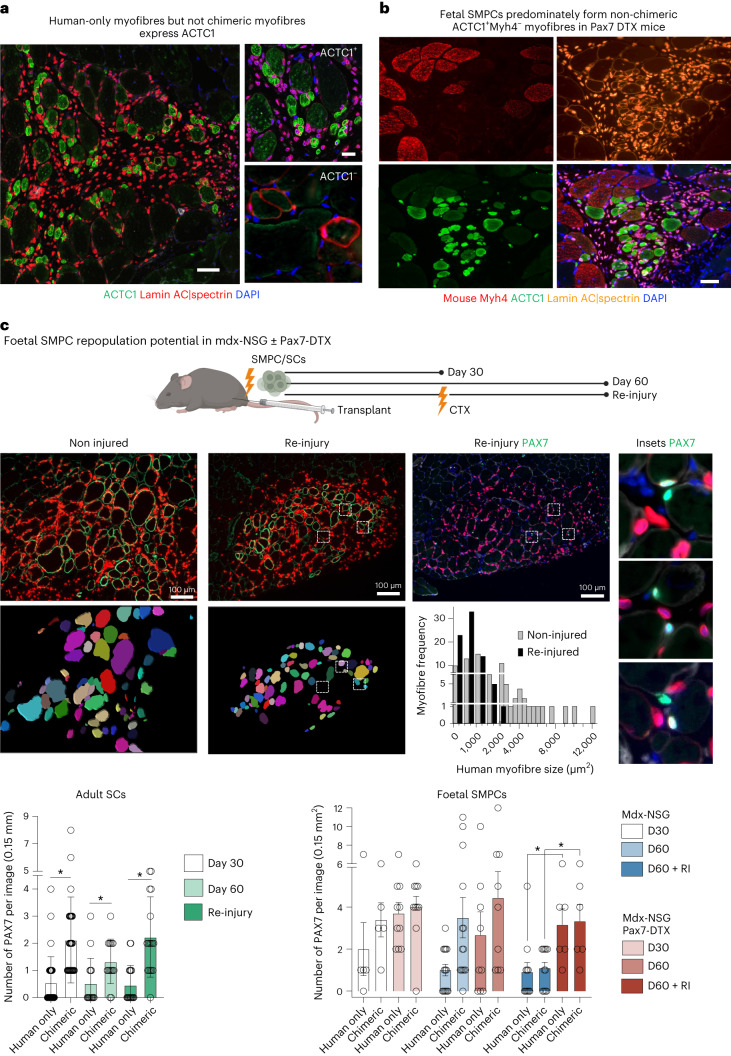
ACTC1 is expressed by human-only myofibres and increased in Pax7-ablated mice, enabling improved SMPC repopulation after injury. **a**, SMPC engraftment after 30 days show that human-only myofibres, but not chimeric myofibres express ACTC1 (green). *N* = 4 engrafted tissues. Scale bar, 100 μm. **b**, Human-only myofibres, but not chimeric myofibres, express regenerative signature as shown by ACTC1. SC niches form near small regenerating myofibres, which enables PAX7 cells to remain. Scale bar, 100 μm. Mouse TA express high levels of Myh4, which is not expressed by human, enabling further identification of human-only and chimeric myofibres. ACTC1 expression is reduced in chimeric Myh4^+^spectrin^+^lamin AC^+^ myofibres. **c**, Cartoon of repopulation experiments is shown. Top: representative images show fibre size in no injury or after re-injury (dystrophin (green), lamin AC/spectrin (red) or PAX7 (green). Insets show PAX7 location near other PAX7 foetal SMPCs. Fibre size is quantified using Imaris in non-injured or re-injured muscle. Bottom: graphs quantify mean ± standard deviation repopulation ability after re-injury, and dots represent individual images used for quantification; 10–15 images per sample, *N* = 3 adult, *N* = 4 foetal biological replicates per timepoint. PAX7^+^ SMPC/SCs and total human nuclei are quantified from the same image sets. *t*-test, *P < 0.05 between PAX7^+^ SMPC/SCs in chimeric or human-only myofibre niches. Data show that foetal SMPCs cannot repopulate after re-injury, but adult SCs can repopulate in mdx-NSG mice; however, in Pax7-ablated mdx-NSG mice in re-injury (D60 + RI) foetal SMPCs can repopulate both chimeric and human-only myofibres. *t*-test, *P < 0.006. Source data

As the *Actc1*^*+*^ gene was the highest-expressed candidate found in our snRNA-seq data in regenerating mouse satellite cell niches, we evaluated if ACTC1 shares common functions with the human-only myofibres supporting PAX7^+^ SMPC engraftment. ACTC1 is also known to be expressed during foetal skeletal muscle development^48^, which further supported the likelihood that ACTC1 would be important in the emerging niches formed by hPSC SMPCs. Indeed, we found that human-only myofibres from hPSC and foetal SMPCs strongly expressed ACTC1 following engraftment, but in myofibres that we identified as chimeric, ACTC1 was not expressed at 30 days (Fig. 5a and Extended Data Fig. 6b). To further demonstrate that ACTC1 was expressed by human-only myofibres, but not chimeric myofibres, we took advantage of mouse-specific myosin expression Myh4 in tibialis anterior (TA), which human skeletal muscle does not express. Staining confirmed the presence of chimeric myofibres (lamin AC^+^spectrin^+^Myh4^+^) and human-only myofibres (ACTC1^+^Myh4^−^), in which the smallest human myofibres had the greatest ACTC1 expression (Fig. 5b). ACTC1^+^ human myofibres were more prominent when engrafted in mdx-NSG Pax7-DTX mice, and moreover adult SCs now predominately formed ACTC1^+^ human-only myofibres in Pax7-ablated mice, suggesting that, when mouse regeneration is impeded, human SC niche formation initially occurs with regenerating human-only myofibres (Extended Data Fig. 6c).

**Extended Data Fig. 7:**
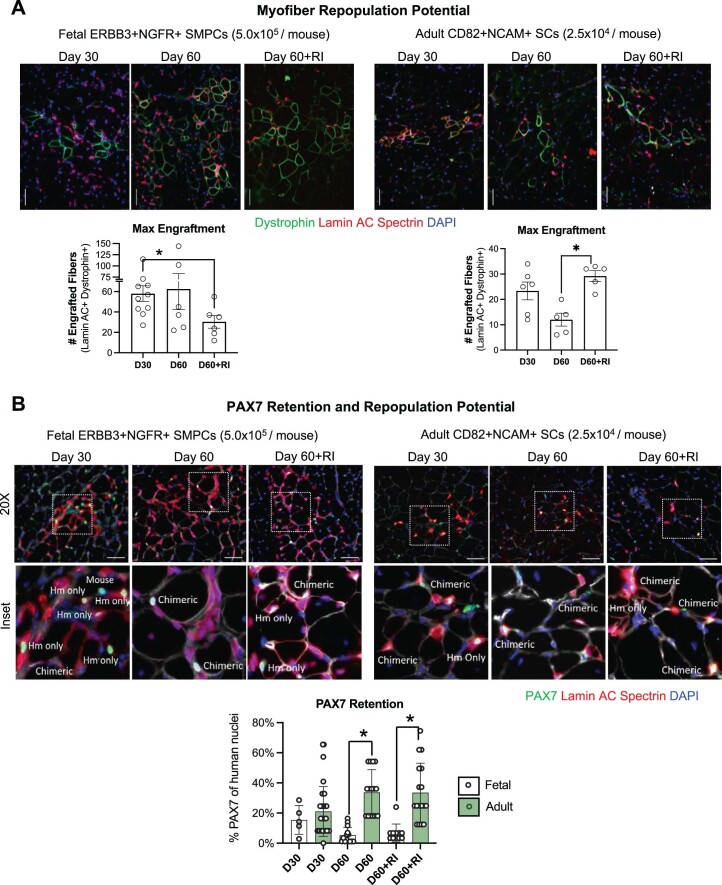
Repopulation potential by adult stem and fetal progenitor cells in mdx-NSG mice. Related to Fig. 5b. **a**. Myofiber formation of engrafted fetal SMPCs (500,000/injection, N = 6) and adult SCs (30,000/injection, N = 10). Graph shows mean ± SEM max number of engrafted myofibers, T-test, * = *P* = *0.02.* **b**. Representative images (**top**) and quantification (below) show mean ± SD of PAX7+ repopulation potential from fetal SMPCs and adult SCs at 30 days, 60 days * = *P < 0.0001*, and after reinjury (day 60+RI) * = *P < 0.0002*. Inset shows representative locations of human (PAX7+LaminAC+) or mouse (PAX7+LaminAC-) muscle cells relative to chimeric or human-only myofibers.

Since we found that SC niche formation is predominantly occurring near human-only fibres in mdx-NSG Pax7-DTX mice we set out to evaluate whether these niches were functional. The hallmark in vivo assay for evaluating true muscle stem cell potential is the re-injury assay, in which PAX7 cells must regenerate new myofibres as well as maintain the stem cell pool^12^. After 30 days, engrafted muscles are re-injured using CTX, which damages myofibres but preserves PAX7 cell regeneration ability^49^. As controls for re-injury, day 30 and uninjured day 60 engrafted tissues are used. We found human adult SC and foetal SMPCs were able to form dystrophin^+^ myofibres and retain some PAX7^+^ cells at all timepoints, but engraftment dynamics were very different across mouse models (Fig. 5c and Extended Data Fig. 7a). In mdx-NSG mice, adult SCs where able to sustain PAX7^+^ SC numbers in chimeric niches following injury, but foetal PAX7^+^ SMPCs decreased by >2-fold, and the ratio of PAX7 foetal SMPCs in chimeric niches decreased by 43% (Fig. 5c). After re-injury, 1 in 3 transplanted adult SCs expressed PAX7, whereas only 1 in 15 foetal SMPCs expressed PAX7 (Extended Data Fig. 7b). In contrast, in the mdx-NSG Pax7-DTX mice, we found after re-injury there was a notable increase in the ability of foetal SMPCs to repopulate both human-only and chimeric niches (Fig. 5c). We also found several instances where two or more PAX7^+^ foetal SMPCs were within 5 µm of each other, which is suggestive of daughter foetal SMPCs derived from a common parent following re-injury (Fig. 5c). These data show that the increase in ACTC1^+^ human-only myofibres in the mdx-NSG Pax7-DTX mice can support SC niche formation and repopulation after re-injury.

### Spatial analysis identifies key emerging niche pathways

**Fig. 6:**
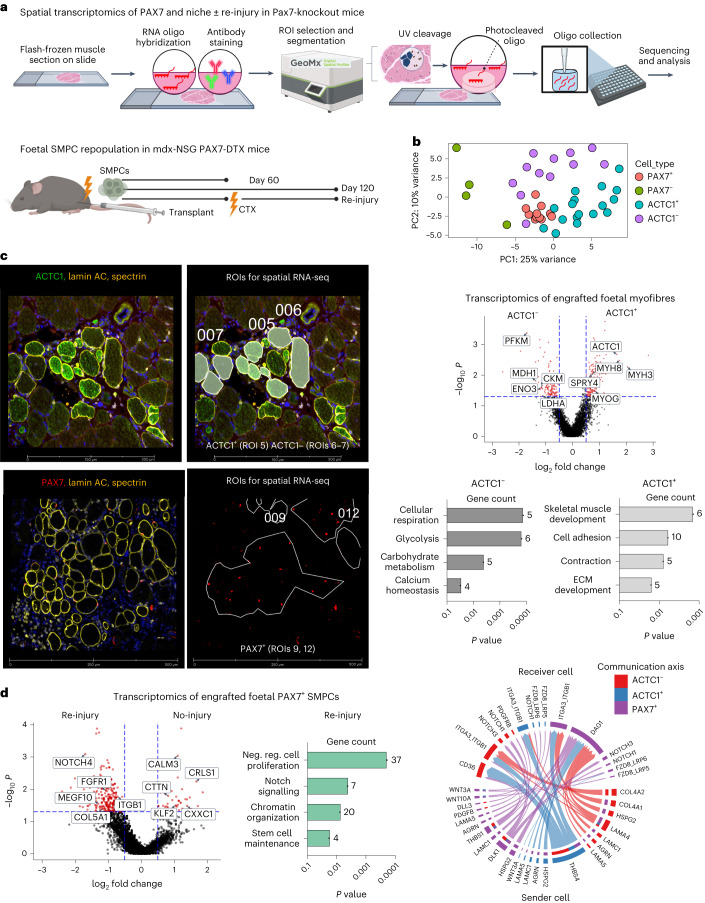
Spatial transcriptomics identifies interaction of ACTC1^+^ myofibres with PAX7 SMPCs in the emerging niche. **a**, Cartoon workflow of spatial transcriptomics on engrafted human/mouse tissue. Photocleavable RNA oligo probes are collected on ROIs for sequencing. Tissues used for spatial RNA-seq included foetal SMPCs engrafted in PAX7-ablated (or DTX) mdx-NSG mice for 60 or 120 days, and re-injury with CTX at 60 days. **b**, PCA plot showing clustering of engrafted human cells (PAX7^+^, PAX7^−^) and myofibres (ACTC1^+^, ACTC1^−^). Each dot represents a sequenced ROI (*N* = 43). **c**, Top left: images taken on GeoMX of day 60 engrafted foetal SMPCs show ACTC1^+^ and ACTC1^−^ myofibres. GeoMx applied masks to ROIs used for sequencing. Right: volcano plot of ACTC1^+^ and ACTC1^−^ myofibres. Red dots represent differential expressed genes, and selected key genes are shown. For all volcano plots differential gene expression (DGE) between ROIs was determined using GeoMx linear mixed model (LMM) statistical tests with Benjamini–Hochberg (BH) correction *P* < 0.05. Bar graphs show Gene Ontology of selected pathways upregulated by ACTC1^+^ and ACTC1^−^ myofibres. Bottom left: PAX7 in the re-injury group and regions used for selecting single PAX7 cells for sequencing. **d**, Volcano plot of human foetal PAX7^+^ SMPCs after re-injury or no-injury 120 days after engraftment. Red dots represent differentially expressed genes, and selected key genes are shown. Graphs show Gene Ontology of foetal PAX7^+^ SMPCs undergoing repopulation after re-injury of selected pathways related to the SC niche comparisons between two groups. For comparing different cell types or treatments to generate volcano plot data, LMM statistical tests with BH correction were performed. Neg. reg., negative regulation. Right: CellChat analysis show predicted sender (bottom) and receiver (top) ligand and receptor interactions between foetal PAX7^+^ SMPCs and ACTC1^+^ and ACTC1^−^ myofibres from spatial RNA-seq of engrafted tissues. Source data

To better define the ACTC1^+^ niche supporting human PAX7 cells, we optimized spatial RNA sequencing (RNA-seq) and evaluated the niche in both long-term engraftment and after re-injury (Fig. 6a). To do this, we used the Nanostring GeoMx platform, which enables cell type and location specific analysis of gene expression at single antibody resolution. GeoMx allows for region of interest (ROI) selection and segmentation on PAX7 cells and ACTC1^+^ and ACTC1^−^ myofibres (Supplementary Tables 3 and 4). In brief, we engrafted foetal SMPCs for up to 4 months and induced an injury in a subset of engrafted mice midway at 2 months. We then mounted flash-frozen muscle sections on microscope slides, hybridized a whole transcriptome human RNA oligo library onto the muscle, and stained with morphology markers ACTC1, PAX7, lamin AC and spectrin to identify human muscle engrafted in mouse. After ROI selection and segmentation, the RNA oligos are photocleaved and collected for sequencing (*N* = 44 ROIs, Extended Data Fig. 10, deposited as GSE243875). Principal component analysis (PCA) found distinct clustering of cell types including ACTC1^+^ and ACTC1^−^ myofibres (Fig. 6b), and our data confirmed a robust repopulation ability by foetal SMPCs in mdx-NSG Pax7-DTX mice that was sustained through 4 months (Fig. 6c). At the time of re-injury, we identified unique gene signatures in the ACTC1^+^ myofibres, which included increased embryonic and foetal myosins, ECM development and cell adhesion. In contrast the ACTC1^−^ myofibres had higher expression of metabolism genes and maturation genes. We additionally performed DEG analysis of PAX7 foetal SMPCs after repopulation versus PAX7 foetal SMPCs that remained over a 4-month time period (Supplementary Table 5). Foetal PAX7 SMPCs that had undergone repopulation expressed many genes that could be unique candidates associated with new niche formation including *NOTCH4*, *ITGB1*, *FGFR1* and *MEGF10* as well as stem cell maintenance, chromatin organization and negative regulation of cell proliferation (Fig. 6c,d). We then used CellChat analysis to predict receptor–ligand interactions between the ACTC1^+^ myofibres and the PAX7 foetal SMPCs identified potential candidates associated with emerging niches including robust association of DAG1 on PAX7 cells and LAMA5 as well as LAMC1 on ACTC1-positive myofibres. Interestingly, THBS4 was a top candidate on ACTC1^+^ myofibres interacting strongly with receptors on PAX7 cells including integrins and with other myofibres through CD36, a regulator of lipid metabolism and fatty acid uptake (Fig. 6d). Thus, spatial analysis revealed several potential interacting pathways supporting emerging niche formation in vivo in addition to ACTC1.

### ACTC1^+^ myofibres are essential for PAX7 in emerging niches

**Fig. 7:**
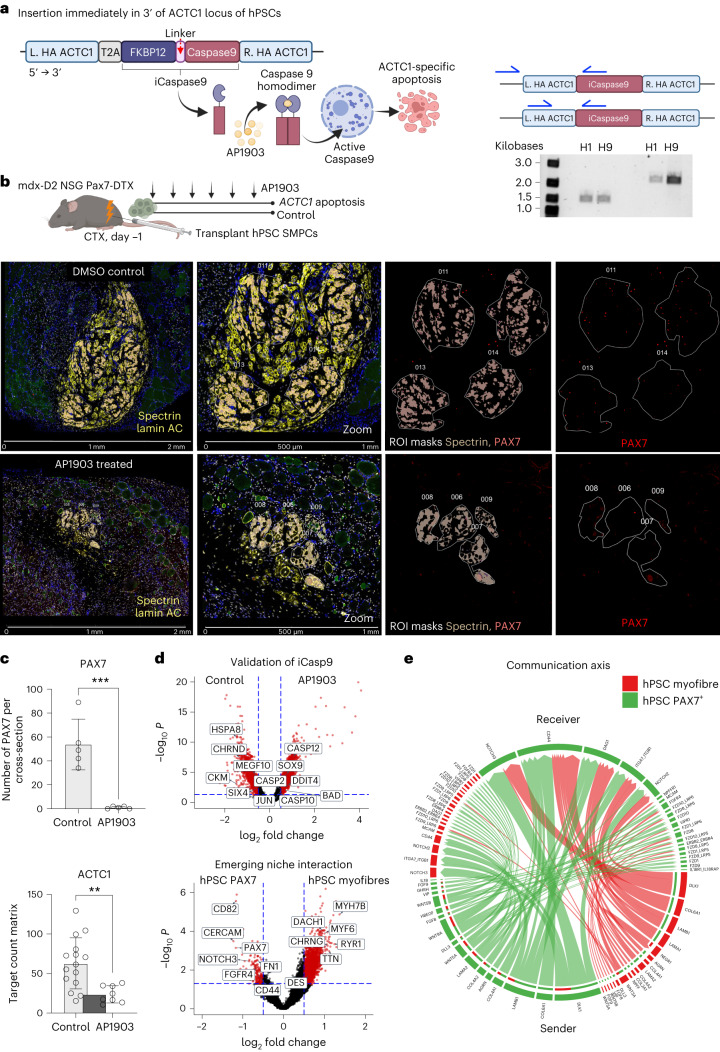
ACTC1 is essential for emerging niche formation and support of hPSC PAX7 SMPCs. **a**, Cartoon showing iCaspase9 vector (iCasp9) insertion immediately in 3′ of ACTC1 locus of hPSCs. AP1903 causes iCasp9 activation and gene-specific cell death. PCR products show insertion of iCasp9 across two hPSC lines (H1 and H9). **b**, Top: Pax7-DTX (ablated) Mdx-D2-NSG mice were treated with TMX, and hPSC SMPCs transplanted into Pax7-ablated tissues. Mice were treated six times every two weeks with AP1903 over 90 days, *N* = 5 cross sections. Left: images taken on the GeoMx of 90-day engrafted hPSC SMPCs show regions of transplantation with or without AP1903 treatment. Zoom shows morphology markers and masked ROI overlays. Single PAX7 cells and myofibres were collected for spatial RNA-seq. **c**, Graph show mean ± standard deviation of PAX7 counts from cross-sectional area and a decrease in AP1903-treated mice; *t*-test,***P = 0.0005, *N* = 5 cross sections. ACTC1 expression counts were obtained from Q3 normalized outputs from ROIs on GeoMX; **P < 0.003, *N* = 13 untreated, *N* = 8 AP1903. **d**, Top: volcano plot of all human ROIs in control and AP1903-treated group highlight increased caspase activity in AP1903-treated mice. Bottom: volcano plot in control mice shows genes upregulated hPSC PAX7^+^ SMPCs compared with hPSC myofibres. Red dots represent differentially expressed genes, and selected key genes are shown. For comparing different cell types or treatments to generate volcano plot data, linear mixed model (LMM) statistical tests with Benjamini–Hochberg (BH) correction were performed. **e**, CellChat analysis show predicted sender (bottom) and receiver (top) ligand and receptor interactions between hPSC PAX7 SMPCs and hPSC myofibres from spatial RNA-seq of engrafted tissues.

**Extended Data Fig. 8:**
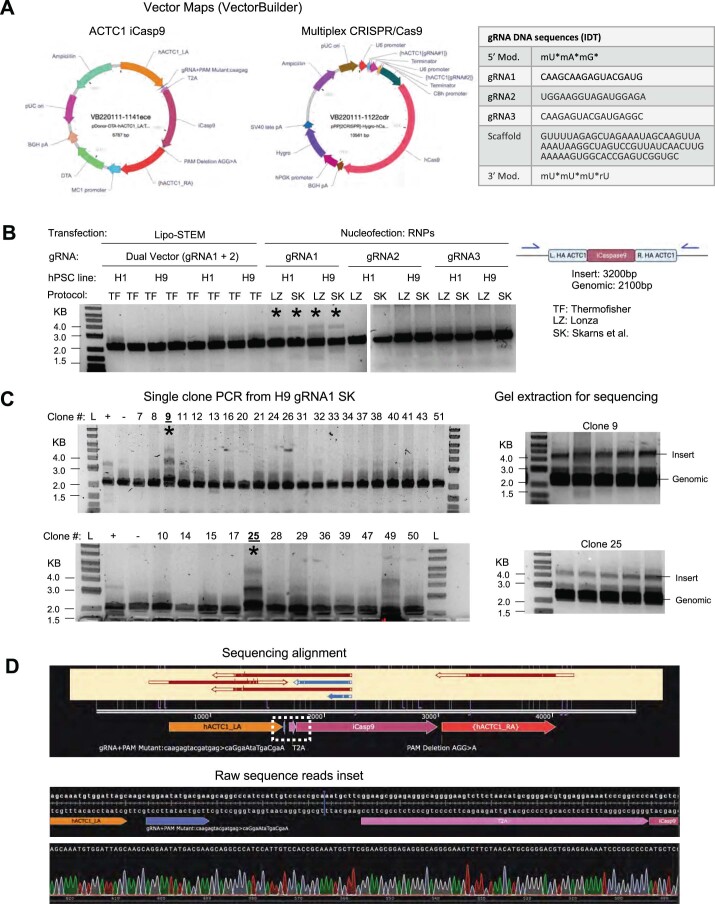
Generation of an inducible apoptosis ACTC1 “kill switch” hPSC line. Related to Fig. 7a. **a**. Vector Maps of ACTC1 iCasp9 and Multiplex CRISPR/Cas9 (VectorBuilder) used for transgene insertion. Table of gRNA sequences, tracer sequence, 3′ and 5′ modifications for improved RNA stability were purchased from IDT. **b**. Gel shows all conditions used to knockin iCasp9 vector into parental H1 and H9 lines, N = 17. Lipo-Stem protocol from TF: Thermofisher, and nucleofection protocols from LZ: Lonza and SK: Skarns et al., were used. 5′ genomic DNA to 3′ genomic DNA spanning the region with the knockin was PCR’d. Asterisk indicates correct band size. **c**. After FACS sorted single cell plating and clonal expansion, Clones were collected and PCR’d for iCasp9 insertion, N = 108. Two heterozygous clones (C9 and C25) were identified (asterisk). Clones were then PCR’d for sequencing. **d**. Sequencing results (Retrogen) of parental, C9, and C25 clones show 100% alignment to expected sequence. Inset shows DNA sequence spanning the 3′ end of ACTC1 gene, T2A readthrough, and start of the iCasp9 transgene.

To definitively prove that ACTC1 myofibres are critical for supporting PAX7 cells, we followed recent approaches that have introduced an inducible suicide gene in hPSCs^50,51^. HPSCs are uniquely able to be genetically manipulated while retaining pluripotency and ability to differentiate into downstream lineages. We engineered an inducible apoptosis system that included a FKBP12 linked to a caspase 9 (iCasp9) and a self-cleaving T2A peptide immediately following a homology arm of the last 3′ base pair in the *ACTC1* gene (Extended Data Fig. 8a). Treatment with the bio-inert small molecule AP1903 induces cross-linking of the drug-binding domains of this chimeric protein, which in turn dimerizes caspase 9 and activates the downstream executioner caspase 3 molecule, resulting in gene-specific cellular apoptosis^52^ (Fig. 7a). To determine the optimal conditions for inserting iCasp9 by homology-directed repair, we tested 17 conditions in H9 and H1 hPSC lines including 3 guide RNA (gRNA) targets, lipo-STEM and nucleofection, and compared homology-directed repair protocols described by Skarnes et al., to the protocol provided by Lonza (ref. ^53^ and Extended Data Fig. 8b). We confirmed the presence of inserted iCasp9 in both H9 and H1 lines following nucleofection (Fig. 7a) and proceeded to single-cell cloning in 384 wells. Of these, 108 clones survived, and we found 2 clones that contained a heterozygous insertion for iCasp9. We performed sequencing to confirm an exact match for our engineered iCasp9 vector within the *ACTC1* locus (Extended Data Fig. 8c).

**Extended Data Fig. 9:**
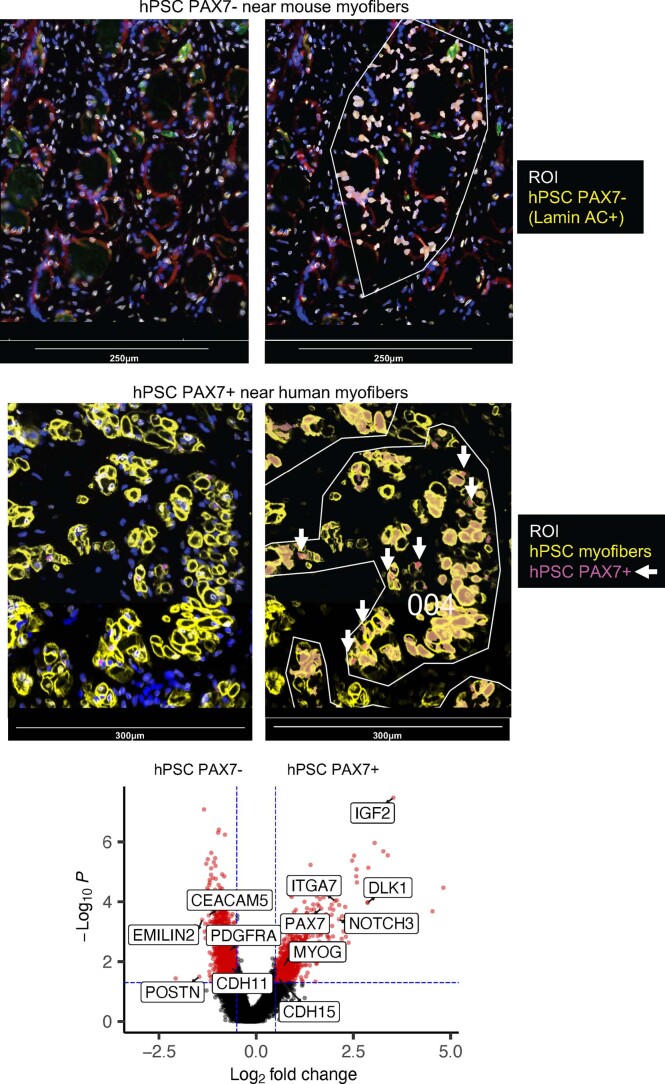
Cell fate of engrafted hPSC Lamin AC+ PAX7- cells in the absence of ACTC1+ myofibers. Related to Fig. 7b. Images taken on the GeoMx of 90 day engrafted PAX7+ hPSC SMPCs and PAX7− hPSC SMPCs with or without AP1903 treatment. Masked ROI overlays (arrows indicate PAX7) show cells that were collected for spatial RNA sequencing. Volcano plot of PAX7+ and PAX7− human cells. Red dots represent differential expressed genes, and selected key genes are shown.

We next performed directed differentiation of ACTC1-iCasp9 hPSCs to generate SMPCs for engraftment. We used Pax7-ablated mdx-D2 NSG Pax7-DTX mice as these mice readily enable human-only myofibre formation. Two weeks following engraftment, mice were treated with AP1903 (10 μM in 100 μl saline) or control (0.1% dimethyl sulfoxide in saline) every two weeks for 90 days. Similar to previous results, in non-treated mice we found hundreds of hPSC myofibres that were able to engraft over approximately 6 mm^3^ of mouse TA, and we counted up to 90 PAX7^+^ hPSC SMPCs associated with hPSC myofibres within a 1.5-mm^2^ region of tissue (Fig. 7b and Extended Data Fig. 9a). AP1903 treatment induced a ∼90% reduction in hPSC myofibres, as we found fewer than 20 myofibres per cross section in treated mice. Remarkably the apoptosis of ACTC1^+^ myofibres resulted in an almost complete elimination of PAX7 cells in the AP1903-treated mice. We counted a total of 269 human PAX7 cells in five regions of control mice and only 5 PAX7 cells within the same engrafted regions of AP1903-treated mice (Fig. 7b,c). However, in the AP1903-treated groups there were hundreds of remaining lamin AC^+^ cells that were neither myofibres nor PAX7^+^.

We further validated the iCasp9 system using GeoMx spatial sequencing on engrafted hPSC muscle cells with and without AP1903 treatment (ROI, *N* = 59). Spatial analysis showed an abundance of cell death-related pathways including several upregulated caspase genes in the AP1903-treated muscles but not in control muscles (Fig. 7c,d and Supplementary Table 6). We compared the remaining hPSC lamin AC^+^ cells (called hPSC PAX7^−^) with hPSC PAX7^+^ cells, and these expressed genes enriched in non-stem cell or non-myogenic related cell fate change including *POSTN*, *CDH11* and *PDGFRA*, suggesting that in the absence of human myofibres these cells change fate to other mesoderm lineages, whereas the PAX7^+^ cells expressed key stem cell genes such as *ITGA7*, *NOTCH3* and *CDH15* (Extended Data Fig. 9 and Supplementary Table 7). To better define how the hPSC SMPCs are interacting with the emerging fibres we compared the PAX7 cells with the hPSC myofibres in vivo again using spatial RNA-seq. We also discovered that key stem cell signalling and adhesion pathways are turned on demonstrating maintenance of stem cell fate in this model when ACTC1-positive fibres are present, indicating support of human PAX7 cells by these emerging niches (Fig. 7d). To verify this, we performed CellChat analysis and found that hPSC myofibres send several signals through embryonic laminins and delta ligands (*LAMA1* and *DLK1*) that are predicted to interact with receptors (*NOTCH3*, *FGFR4*, *DAG1*, *ITGA7* and *CD44*) on the hPSC PAX7^+^ SMPCs (Fig. 7e and Supplementary Table 8). These identified key stemness pathways are turned on, which could be key to supporting hPSC PAX7 cells in emerging niches.

## Discussion

The ability to efficiently support human PAX7 cells after engraftment remains a major roadblock to translating cell therapies for skeletal muscle. We demonstrate that the formation of human-only myofibres following transplantation is a key source of niche emergence from transplanted human cells, which has previously been overlooked. Human-only myofibres have a 50-fold better ability to support transplanted PAX7 cells and can be identified by expression of the foetal skeletal muscle actin isoform ACTC1. The ACTC1^+^ hPSC myofibres form niches that phenocopy late-stage foetal myogenesis, which enables the generation of human niches that support PAX7 cells from hPSCs. To demonstrate that ACTC1^+^ myofibres are key to supporting stemness from PAX7^+^ hPSC SMPCs, we developed iCasp9 suicide hPSC lines where we took advantage of the power of hPSCs with genetic engineering to evaluate this question. We demonstrate that ablation of the ACTC1 myofibres results in substantial PAX7 loss. Using multiple functional studies, we conclude that transplanted human SMPC/SCs form niches using skeletal muscle regeneration pathways and do not appear to ‘home’ to empty niches like the haematopoietic stem cell system^54^. We show reciprocation between the myofibre and stem cell; when mouse Pax7^+^ SCs are ablated, the regenerative Actc1^+^ myofibres are lost, and conversely when ACTC1^+^ emerging myofibres are eliminated, the PAX7 hPSC SMPCs are lost. We also have identified unique gene signatures including ligands and receptors found on foetal and hPSC myofibres that may support de novo niche formation and could improve our ability to translate human PAX7 cells for cell and regenerative therapies.

Our new engraftment model specifically eliminated mouse SCs to evaluate SMPC niche formation in the absence of SCs. Interestingly, in Pax7-ablated mice we found increased numbers of PAX7^+^ hPSC and foetal SMPCs, but rather than residing in empty chimeric mouse niches, SMPCs increased association with ACTC1^+^Myh4^−^ human-only myofibres at 30 days. We also found that PAX7^+^ foetal SMPCs and adult SCs now predominantly formed niches with ACTC1^+^ human myofibres in Pax7-ablated mice, suggesting that in this environment PAX7 SMPCs/SCs prefer association with regenerative myofibres even though our ablation and injury model still contained hundreds of mouse myofibres (Extended Data Fig. 4c). We show that foetal and hPSC SMPCs can maintain PAX7^+^ expression for several months in vivo when associated with ACTC1^+^ myofibres, and when re-injury is applied after 60 days foetal PAX7^+^ SMPCs are now better able to engraft into both human-only and chimeric niches, suggesting association with these niches enables an increase in functional maturation.

Our approach is a more precise ablation model compared with long-standing practices in the field, which has removed competition with the endogenous SCs by irradiation aiming to increase niche occupancy from engrafted donor human or mouse cells. Muscle irradiation studies are confounded by precise dosing and timing regimens required, and high doses of radiation reduce donor mouse-to-mouse engraftment^55–57^. In our hands and others, irradiation did not improve human muscle cell engraftment into mouse^58^. Further, irradiation damages cells in the microenvironment, which orchestrates muscle regeneration and supports SC niche formation^59–61^, and studies have not fully investigated the extent to which radiation regimens affect the myofibre regenerative response. We also cannot rule out that the formation of human-only myofibres will be increased close to the injection site, and therefore would increase the probability of local association with human PAX7 cells. However, this finding is also interesting and could inform on cell numbers needed to support PAX7 SMPCs upon engraftment and the need for multiple injections per muscle.

To understand the mechanisms of failed regeneration and niche formation in Pax7-ablated mice, we performed snRNA-seq in Pax7-ablated and control mice with and without injury. Similar to work from several groups^39–41^, we show that loss of Pax7 cells led to severe muscle regeneration defects in acute-injury models. Of the remaining myofibres in the Pax7-ablated mouse muscle, our sequencing data find increased numbers of highly glycolytic *Car3*^*+*^ myofibres, but most striking was the reduction of the regenerative *Actc1*^*+*^ mouse myofibres marked by several genes involved with sarcomere assembly (*Tmod1*, *Myhpb3* and *Tnnt3*), which suggests that niche formation occurs in parallel with construction of sarcomeres. Notable niche genes identified in the *Actc1*^*+*^ myofibres that interact with laminin include *Itgb1pb2*, which binds integrins expressed by SCs during niche formation^62^, and *Tmem38a*, which regulates heterochromatin and repositions select genes to the nuclear periphery of the myofibre, which happens to include key regulators of SC migration such as Vcam1. It also brings in Nidogen, a gene involved in repositioning of laminin binding and that we found expressed by human foetal and hPSC myofibres in vivo^63^.

Although it is well understood how mouse regeneration occurs after injury, this work develops a human model to study regeneration leading to new emerging niche formation in vivo. We took advantage of spatial RNA-seq to investigate differentially expressed genes between ACTC1^+^ and ACTC1^−^ myofibres and ligand–receptor pairs interacting with genes expressed on foetal SMPCs. One of the unique identified pathways expressed on engrafted ACTC1^+^ foetal myofibres was Thrombospondin 4 (THBS4), which enhances attachment of dystrophin–glycoprotein and integrin complex to stabilize the muscle membranes^64^ and is predicted to interact with ITGB1 expressed on foetal SMPCs. We also performed spatial RNA-seq on engrafted hPSC SMPCs and myofibres, and identified key in vivo niche genes including CD44 expressed by PAX7+ hPSC SMPCs which is activated during regeneration and foetal development^65^, and is predicted to interact with Collagen 6 expressed by hPSC myofibres, which promotes autonomous niche formation through ECM production^66^. Other key identified interactions in our analysis that may also be critical to supporting PAX7^+^ SMPCs include multiple NOTCH signalling pathways that are sending and receiving in both directions from hPSC SMPCs and myofibres. We also found Dystroglycan 1 (DAG1) on hPSC SMPCs interacting with laminin 111 (LAMA1) expressed by hPSC myofibres. One caveat to the use of GeoMx for spatial RNA-seq is the close, but not perfect, precision of morphology marker segmentation, and we do find some muscle genes in the PAX7 SMPC datasets due to their close spatial proximity demonstrating further validations will be required. To avoid this, the datasets we focused on comparing hPSC SMPCs with myofibres to identify enriched genes such as *CD82*, *FGFR4* and *CERCAM*, and these may represent key targets to modulate for improved support of PAX7 cell engraftment in future studies.

The significance for identification of the ACTC1^+^ myofibres for initiating niche formation could have both basic and therapeutic applications. Many labs have highlighted that both regenerating adult myofibres and developing foetal myofibres support PAX7 cell numbers at orders of magnitude greater per unit area than adult myofibres in homeostasis^33,67^. It would be interesting to determine whether myofibres in homeostasis could support additional PAX7 cells if stimulated by myofibre genes involved in emerging SC niche formation. *Actc1* has previously been shown to be highly expressed in foetal skeletal muscle^68^, and overexpression of *Actc1* has been used to rescue defects in skeletal actin myopathies that increased the expression of regenerating markers such as embryonic myosin^69^. Similarly, we found mouse SCs during regeneration also clustered near newly formed Actc1^+^ myofibres. Looking to other stem cell niche systems such as dental pulp^70^ and hair follicle^71^, stem cell density in the niche is far greater than in skeletal muscle, suggesting the upper limit to stem cell density in niches found in biological systems could be increased. Regulation of the mouse Pax7 cell number set point has been demonstrated via Wnt4 knockdown^72^, and increasing the SC number set point could have exciting implications for muscle regeneration and establishing niches from hPSCs.

Our results highlight that SMPCs that do not associate with human myofibres lose PAX7 expression. Loss of stemness also occurs for mouse SCs when displaced from the myofibre niche^73^. Thus, myofibres are important to support the stem cell state. In the absence of myofibres in Myf5/MyoD double-null mice, satellite cells can adopt mesenchymal or alternative somatic lineages after injury, but the double-null satellite cells were retained when myofibres were present and uninjured. This observation suggests that myofibres contribute to maintaining the identity of satellite cells having the ability to adapt non-myogenic cells^74,75^. Similarly, using iCasp9 in human ACTC1 myofibres, we found that hPSC SMPCs adopted alternative cell fates when not associated with myofibres (Extended Data Fig. 9). PAX7^−^lamin AC^+^ cells expressed high levels of mesenchymal (*PDGFRA* and *POSTN*) and mesodermal (*CDH11*) genes. Both the lack of niche association and the immaturity of hPSC SMPCs may also result in increased plasticity, similar to work showing that foetal SMPCs have plasticity to form both bone/muscle without niches^27,76^.

**Extended Data Fig. 10:**
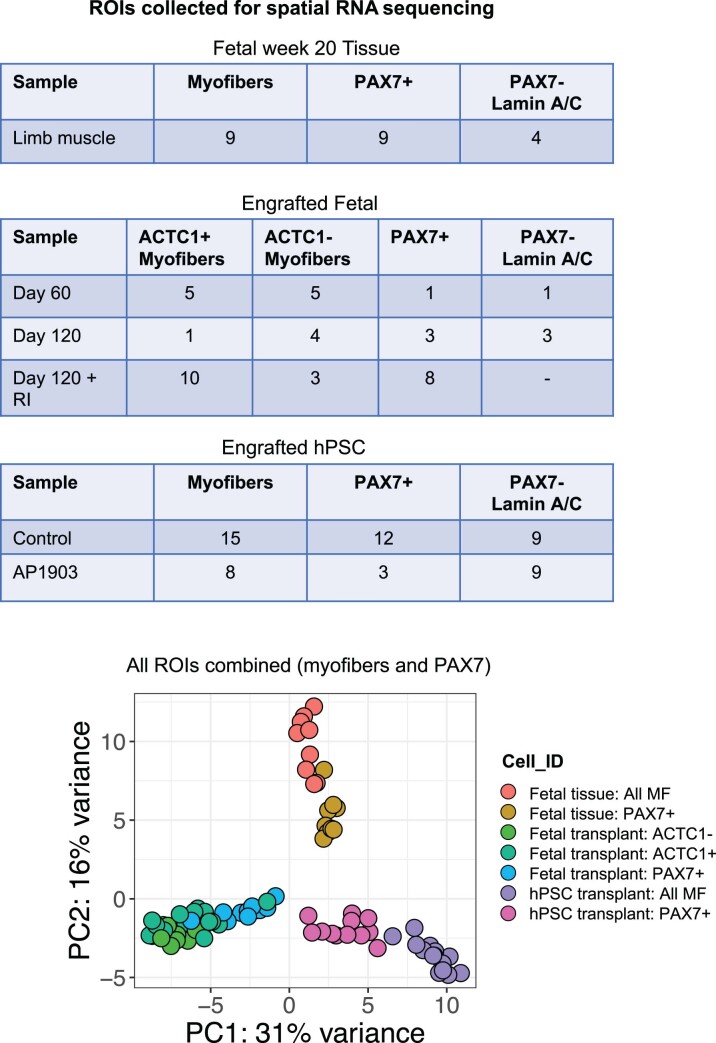
Regions of interest (ROIs) and combined data collected for spatial RNA-Sequencing. Related to Figs. 6 and 7. **a**. Table shows total number of ROIs used for spatial RNA seq from week 20 fetal tissues (N = 22), engrafted week 20 fetal SMPCs in mouse TA (N = 42), and engrafted hPSC SMPCs in mouse (N = 59). ROIs are categorized by morphology markers at time of collection. **b**. PCA plot comparing spatial RNA seq three samples (week 20 fetal tissues, hPSC transplant, and fetal transplant) and two groups (PAX7+ SMPCs, and myofibers). As fetal engrafted myofibers contained ACTC1+ and ACTC1− myofibers, these further separated. Plot show that PAX7 cells across tissues align more closely than do myofibers across tissues.

The in vivo microenvironment has been shown to improve maturation of mouse embryonic stem cells following overexpression of Pax3 or Pax7 (iPAX)^77^ and hPSC SMPCs in vivo^78^, but all are largely foetal and not equivalent to quiescent adult SCs. Recent work has also shown that maturation can be enhanced by epigenetic modulation in other stem cell systems^79,80^. We evaluated hPSC SMPCs in vivo compared with engrafted foetal or adult SCs and tissues, and our work suggests that hPSC SMPCs do mature in vivo from an embryonic week 7–12 to a later foetal week 17–20 stage, but do not fully mature to quiescent adult SCs. How human SMPCs become quiescent SCs in the niche is an exciting direction that could require lineage tracing to follow SC and niche dynamics over time in vivo. Interestingly, we find that the myofibre populations between engrafted foetal and hPSC myofibres and foetal tissues are more dissimilar than are the PAX7^+^ cells (Extended Data Fig. 10), and thus the myofibres may be one of the most important targets for PAX7^+^ SMPC maturation, including approaches to enhance innervation.

In summary, we developed a new muscle stem cell ablation mouse model compatible with engraftment and demonstrated that emerging niche formation from hPSCs forms similar to the process of regeneration. This work has global implications for cell therapies and provides a new model system to study how to support human regeneration and niche emergence. Further, this new system could provide a platform to screen for new regulators of niche formation and the key regulators of stem cell–niche interactions supporting PAX7 cells in vivo.

## Methods

Our research complies with all relevant ethical regulations. All hPSC research was approved by the hSCRO committee (2006-009-17), all biosafety was approved by IBC (BUA-2019-128-012-CR), all animal work was approved by the ARC (ARC-2006-119) and all foetal and human tissue research was approved as exempt by the institutional review board (IRB; 20-000197). All foetal and human tissues came de-identified with no patient information. Consents were obtained according to the ABR consent forms (foetal tissue) and NIH NDRI Tissue repository for human cadaver tissue. There was no participant compensation.

### hPSC culture, directed differentiation into SMPCs and derivation of BLI hPSC lines

To improve myogenic potential, we revised the directed differentiation from previously described protocols^14,20^. In brief, hPSC lines were independently optimized using seeding density and CHIR99021 concentration, and all directed differentiation medium changes were based on morphology and metabolic rate rather than set time points described below. Revisions included addition of transforming growth factor-β inhibitor (TGFβi) in vitro during the final week of myogenic differentiation to improve myotube formation and support SMPCs and addition of insulin-like growth factor-1 (IGF-1) in the final 3 weeks to support SMPC survival and specification. TGFβi was removed from in vivo experiments as previously reported^20^.

All hPSC experiments were performed with ESCRO approval, and hPSCs were obtained from WiCell and the UCLA stemcellcore. hPSC lines used in this study included H9 (WA09, NIH 0062) (Figs. 1, 2 and 4 and Extended Data Figs.1–3 and 6), H9 with a PAX7-GFP reporter^81^ (Figs. 1, 2 and 4 and Extended Data Figs. 1–3 and 6), H9 with a luciferase reporter (Figs. 2 and 4 and Extended Data Figs. 1–4), CDMD 1002 and CDMD 1006-1 (ref. ^19^) (Figs. 1 and 2 and Extended Data Figs. 1–3) and WTC-11 (Coriell, AICS-0054-091) (Figs. 1 and 4 and Extended Data Figs. 1, 2 and 6). To generate the luciferase reporter, an EF1α-LUC-Puro lentivirus was used to infect hPSCs (H9 and H9 PAX7-GFP) in OptiMEM containing protamine sulfate. Three days after infection, hPSCs were selected by progressively increasing the puromycin concentration for 2 weeks. hPSCs were then single-cell dissociated in CloneR and allowed to form clonal colonies. Six colonies from H9 and four colonies from H9 PAX7-GFP were generated and karyotyped (Cell Line Genetics). Normal, non-differentiated colonies were selected (two H9 clones and one H9 PAX7-GFP clone) and sequenced by LAM PCR (Creative Biogene) to determine viral integration sites.

hPSC lines were dissociated in TrypLE and plated at 275,000–475,000 cells per well of a six-well plate in mTESR containing 10 µM ROCK inhibitor for 1 day to allow for recovery (day 1). hPSCs were then treated with 8–10 µM CHIR99021 (Tocris, 4423) in E6 medium for 2–3 days to induce mesoderm formation. E6 medium was used to generate mesodermal Pax3^+^ cells for 7–10 days. StemPro-34 medium containing 10% StemPro-34 supplement, 5 ng ml^–1^ basic fibroblast growth factor (bFGF), 2 mM l-glutamine, 0.45 mM monothioglycerol, 10.7 μg ml^–1^ transferrin and 0.4% penicillin-streptomycin was used to expand Pax3^+^ cells for 6–8 days. E6 medium was then added to cells for 3 days, and 10 ng ml^–1^ IGF-1 was added to the E6 medium for 7–10 days to enable specification of Pax7^+^ cells. N2 medium (1% N2 supplement, 1% insulin-transferrin-selenium (ITS, Gibco), 10 ng ml^–1^ IGF-1 and 0.4% penicillin-streptomycin) was added to cells for 5–7 days, and N2 medium containing TGFβi (SB431542; 5 μM) was added for 5–7 days to differentiate hPSC SMPCs.

At the end of directed differentiation, hPSC SMPCs were prepared for FACS by dissociation in TrypLE and titrated to break apart three-dimensional structures. Cells were filtered through 100-µm meshes, washed in PBS and resuspended in FACS buffer (2% FBS in PBS). hPSC SMPCs were labelled with antibodies against HNK1 (BioLegend, 359603), ERBB3 (BioLegend, 324705) and NGFR (BD Biosciences, 562123) (1.5 µl per 1 × 10^6^ cells) and far-red viability dye (ThermoFisher, L10120; 0.3 µl per 1 × 10^6^ cells) for 45 min at 4 °C and sorted on a BD FACSAria II. Fluorescence minus one (FMO) controls were used for all gating controls. Samples were sorted and cultured in skeletal muscle basal medium-2 (SkBM-2, Lonza).

### Husbandry and generation of mouse strains

To generate immunocompromised mice for human muscle cell engraftment, NOD-SCID-Gamma (NSG) mice (Jackson Laboratory, 005557) were crossed to C57BL/6J (C57; Jackson Laboratory, 000664), mdx C57BL/10 (mdx; Jackson Laboratory, 001801) or mdx-DBA/2 mice. mdx-DBA/2 mice were a kind gift from the laboratory of M. Spencer generated by crossing mdx to DBA/2J mice (Jackson Laboratory, 000671), which are deficient in *Ltbp4* and *Anxa6*. Pups were continually backcrossed to parental C57 or mdx strains for 5–8 generations to create >90% congenic strains while retaining mutant *Il2* and SCID alleles. Genotyping was performed using Transnetyx. All animal work was conducted under protocols approved by the UCLA or UC Irvine ARC in the Office of Animal Research Oversight. Mice were housed in A+-rated barrier facilities in sterile cages with standard 12-h light and 12-h dark cycles.

Congenic homozygous C57-NSG, mdx-NSG and mdx-DBA/2-NSG mice were used for engraftment studies or further crossed to generate Pax7 ablation models. C57-NSG, mdx-NSG and mdx-DBA/2-NSG mice were crossed to Rosa-DTA mice (Jackson Laboratory, 009669) or Pax7-Cre/ERT2 mice (Jackson Laboratory, 017763). Both Rosa-DTA and Pax7-Cre/ERT2 mice were crossed to C57-NSG, mdx-NSG and mdx-DBA/2-NSG strains for 3–5 generations before the start of Pax7 ablation experiments. Congenic homozygous Pax7-Cre/ERT2 males were then crossed to homozygous Rosa-DTA females, and heterozygous F_1_ *Pax7*^Cre/WT^; *Rosa*^DTA/WT^ mice were used for Pax7 ablation studies. Equal numbers of male and female mice were used at random. All mouse experiments were conducted when mice were between 5–10 weeks of age.

### Assessment of mouse Pax7 ablation

Ablation of endogenous SCs was achieved using 400 mg ml^–1^ tamoxifen chow (Envigo) or mice were injected intraperitoneally (i.p.) with 100 µl of 5 mg ml^–1^ dihydroxytamoxifen (Sigma) diluted in 90% peanut oil and 10% ethanol. Pilot studies tested 3, 7 or 14 days of tamoxifen chow and 1, 3 or 5 days of dihydroxytamoxifen i.p. injections (*n* = 3 mice per group). Non-transgenic *Pax7*^Cre/WT^; *Rosa*^WT/WT^ mice were used as controls. Mice were killed immediately following treatment or 5 days after tamoxifen washout. Tissue was dissected and immunostained for Pax7 (DSHB), laminin and DAPI to count satellite cell numbers. Laminin and H&E staining were also used to measure myofibre and muscle cross-sectional area. Multiple tissue sections and images were taken for each mouse. We found that 7 days of chow, or 3 days of i.p. injections, followed by a 5-day washout period was sufficient to ablate up to 90% of satellite cells compared to non-transgenic controls (*n* = 5 mice).

### Engraftment protocol and preparation of hPSC and foetal SMPCs and adult SCs

Human adult tissues were provided irregularly from donor cadavers from NDRI. All foetal tissues were IRB exempt and were obtained from ABR or the UCLA tissue core. Lin^–^ERBB3^+^NGFR^+^ cells from embryonic/foetal weeks 9–20 or Lin^–^CD82^+^NCAM^+^ adult SCs from years 30–60 were enriched from primary tissues using FACS and immediately transplanted into mouse models. Primary SMPCs and SCs were dissociated as described^20^, washed in Hank’s Balanced Salt Solution (HBSS) and concentrated at 1 × 10^4^–1 × 10^6^ cells per 5 µl of HBSS.

After sorting, hPSC SMPCs were cultured and expanded in SkBM-2 plus bFGF for as short a time as possible (2–3 days) before engraftment to prevent loss of PAX7 expression. hPSC SMPCs in culture were dissociated using TrypLE, pelleted at 300*g* and resuspended in HBSS at 1 × 10^6^ cells per 5 µl. This allowed us to obtain at least 1 million cells per mouse for engraftment. Mouse models aged 6–8 weeks were subjected to a muscle injury by intramuscular injection of 50 µl of 10 µM cardiotoxin (Sigma) or 1.2% barium chloride, 24 h before cell injections. Pelleted cells were kept at 4 °C and transported to the UCLA Humanized Mouse Core. Mice were anaesthetized using 2% isoflurane, and 5–10 µl of cells in solution were injected into the TA muscle using Hamilton microsyringes.

For Pax7 ablation studies, mice were given tamoxifen chow for 7 days and allowed a washout period of 5 days before engraftment. On day 4 of washout, mice were given a cardiotoxin or barium chloride injection to induce muscle injury. Non-transgenic mdx-NSG mice served as controls. For some Pax7 ablation studies, we also used MACS-enriched (CD45^–^CD235a^–^) foetal SMPCs. For *Pax7*-knockout studies, after notice was received that adult clinical samples had arrived, mice were given tamoxifen for 4 days with washout for 1 day and adult SCs were expanded in SkBM-2 for 5–7 days before engraftment.

### Quantification of human PAX7 in engrafted samples

Using a microcryotome, 1,200 µm of TA muscle was removed; 20 sections were collected on positively charged frosted microscope slides in 50-µm intervals, and 300 µm of tissue was collected for RNA or western blots. In this manner, we calculated that each quadrant contained 1,300 µm of muscle tissue. Quadrants were collected until the entire TA was sectioned. Thus, we could determine the position of each cross-section for downstream analysis of cell engraftment. Microscope slides were kept in aluminium foil-covered boxes at –20 °C.

To quantify cell engraftment, all human lamin AC^+^spectrin^+^ and human dystrophin^+^ myofibres from at least 11 cross-sections were counted. To calculate the maximum number of engrafted fibres, the cross-section containing the greatest number of human lamin AC^+^spectrin^+^dystrophin^+^ myofibres was averaged with other cross-sections containing the greatest number of human myofibres from all mice within a treatment group (*n* = 3–7). The maximum engrafted human myofibre average ± s.e.m. was then used to compare to other treatment groups by one-way ANOVA with post hoc Tukey’s test for multiple comparisons (*P* < 0.05).

To quantify PAX7^+^ cells, we first identified regions containing human lamin AC^+^ nuclei. We then captured images of all human PAX7^+^ cells in this region at ×20 magnification (0.15 mm^2^; Zeiss Axio-Observer). Data are represented as total PAX7^+^ cells per image, rather than total PAX7^+^ cells per cross-sectional area of a whole tissue, as high-magnification images were required to quantify niche location (that is, within basal lamina) and quantification required multiple fluorescence channels to confirm human PAX7 origin and enabled multiple users to quantify our large image library. At least two independent users quantified data from each high-magnification image to determine the numbers and locations of PAX7^+^ and lamin AC^+^ regions, and thus using the percentages of PAX7^+^ cells was a more robust quantification method.

Slides were co-stained for lamin AC and spectrin to mark engrafted human myofibres and PAX7^+^ cells were designated as associated with chimeric or human-only myofibres from 18 independent image regions (*n* = 5 engrafted mice; Fig. 1). We next identified engrafted lamin AC^+^ locations and categorized these into four regions: (1) interstitial space, within endomysial regions but with few engrafted myofibres; (2) dense lamin AC^+^ regions, with fields of view containing >100 human cells but few myofibres; (3) chimeric myofibres, with greater than half the field of view occupied by chimeric myofibres; and (4) human-only fibres, where the field of view contained >50 human-only myofibres (Supplementary Fig. 2). After identification of regions, a total of 15–18 images containing PAX7^+^ cells from *n* = 3 mice were quantified; statistical analyses were performed using one-way ANOVA. GraphPad v.6.0 software was used to graph and calculate significance at *P* < 0.05.

### Immunofluorescent staining protocol for engrafted muscle tissues

To perform staining, sections on slides were thawed from –20 °C freezers and washed in PBS containing 0.2% Tween-20 (PBST). Slides were fixed in 4% paraformaldehyde (PFA) for 10 min and washed with PBST. Sections were then blocked in 10% goat serum in 0.2% gelatin, 3% BSA and 0.1% Tween-20 for 90 min at room temperature. Sections were immunostained with human-specific antibodies to spectrin (1:80; Leica, NCL-SPEC1), lamin AC (1:120; Vector Laboratories, VP-L550) and dystrophin (1:6; DSHB, MANDYS106) to positively identify engrafted human cells and examine whether they were capable of fusing to restore human dystrophin expression. Sections were alternatively stained with antibodies recognizing human PAX7 (10 µg ml^–1^; DSHB), lamin AC, spectrin and laminin (1:150; Sigma, L9393) to identify human SMPCs and SCs residing in the satellite cell position. Antibodies to ACTC1 (1:100; Sigma, A9357), Myh3 (1:100; DSHB, BF-45), Myh4 (1:100; DSHB, BF-F3) and M-cadherin (1:100; BD Biosciences, 61110) were used to identify regenerative myofibres. Slides were washed and tissues were counterstained and mounted with DAPI (Vector Laboratories) and imaged using a Nikon Eclipse 600 or Zeiss Axio-Observer 1. High-magnification images were obtained on a Zeiss LSM 880 confocal microscope.

### Immunofluorescent staining protocol for paraffin-embedded sections

Muscle tissues dissected from human specimens were placed in 2% PFA overnight. Tissues were taken to the Translational Pathology Core Laboratory (TPCL) at UCLA for embedding. To deparaffinize tissues, slides were immersed in xylene followed by serial dilutions of 100% to 50% ethanol and distilled water. For antigen retrieval, slides were placed in a jar containing sodium citrate and then placed in a hot pressure cooker at low pressure for 10 min. Slides were washed and blocked using 10% goat serum with 3% BSA and 0.1% Tween-20 in PBS for 90 min at room temperature. Primary antibodies were added overnight at 4 °C. Slides were washed, and horseradish peroxidase (HRP)-conjugated secondary antibodies were added for 45 min. After washing, fluorophore-specific TSA working solution (PerkinElmer) was added for 10 min. For multiplex staining, HRP was quenched using H_2_O_2_ for 15 min (Supplementary Figs. 3 and 7). Slides were washed and tissues were counterstained and mounted with DAPI (Vector Laboratories) and imaged.

### Single-nucleus RNA-seq

mdx-NSG Pax7 DTA mice were given tamoxifen chow for 7 days to ablate SCs. After 3 days of washout with normal chow, 50 μl barium chloride was injected into the TA muscles. Non-injured ablated mdx-NSG Pax7 DTA and injured mdx-NSG mice served as controls. Eight days after injury, mice were killed and TA muscles were prepared for single-nucleus RNA-seq. To prepare nuclei, we used the custom-developed ‘Frankenstein’ protocol for nuclear isolation from fresh and frozen tissue and muscle prepared as described by 10x Genomics. Prepared myonuclei were stained with DAPI and taken to the UCLA JCCC flow core, and myonuclei were sorted on the On-Chip FlowCell Microfluidic Chip-Based Gentle Cell Sorter. Nuclei were collected in 10× RT buffer, and the concentration of the nuclei was adjusted to 1,000 nuclei per μl. Following quality control on the D1000 ScreenTape, nuclei were prepared for 2 × 50-bp paired-end sequencing on the NovaSeq 6000 at the UCLA Technology Center for Genomics & Bioinformatics.

A total of 1.43 billion reads on 14,302 nuclei were generated (average of 100,000 reads per nucleus). FASTQ files for each sample were processed independently with the default pipeline in Cell Ranger Single-Cell Software Suite 3.0.2 by 10x Genomics. We used a reference genome built against mouse mm10 (sequence: GRCm38 Ensembl 93). Quality control, normalization, filtering, data visualization and differential expression tests were performed in Seurat (v.3.2.2) (Ref. ^82^) with minimal custom modification to the standard workflow. The datasets were analysed independently and then integrated using the standard Seurat integration workflow for SCTransform-normalized data. For individual datasets, cells expressing fewer than 200 and more than 2,000 genes were filtered out. Moreover, cells with >5% of UMIs mapped to mitochondrial genes were removed. After SCTransform normalization, we performed PCA on the gene expression matrix. Respectively, we used the first 30, 30, 18 and 23 PCs for mdx-NSG mice, mdx-NSG mice 8 days after injury, Pax7-ablated mice and Pax7-ablated mice 8 days after injury for clustering and visualization. Once analysis was performed independently, integration analysis was performed using the Seurat integration workflow with 35 PCs and UMAP resolution of 1.9. A Wilcoxon rank-sum test of IIB.3 compared to gene averages of all populations from single-nucleus RNA-seq, with 0.25 min.pct, 0.25 logfc.threshold, was used to generate functional annotation of IIB.3 myofibre populations.

### ACTC1 knock-in method

To generate an inducible ACTC1-expressing suicide cell line, we used both the H1 and H9 lines for CRISPR-mediated homology-directed repair. For nucleofection, hPSCs were single-cell dissociated and 500,000 hPSCs were nucleofected using an Amaxa 2D Nucleofector (shock program B-016) along with purified spCas9 (IDT), gRNAs (sequences provided in Supplementary Fig. 8) and template ACTC1 knock-in plasmid (6–10 µg; VectorBuilder). For the protocol from Skarnes et al.^53^, we included HDR enhancer (IDT) and cultured cells at 32 °C for 2 days following nucleofection, while cells on the Lonza protocol was placed immediately at 37 °C without HDR enhancer. hPSCs were recovered with mTESR Plus and CloneR (Stemcell Technologies). We also tested LipoStem (ThermoFisher), which was used to lipofect a plasmid containing two gRNAs (sequences 1 and 2) and spCas9 alongside the template ACTC1 knock-in plasmid into small adherent hPSC colonies. When cells reached confluence, hPSCs from all conditions were dissociated and split for isolation of genomic DNA (Zymogen) and cryostorage. Genomic DNA was used to screen for presence of the correct genomic insertion using primers designed to amplify through both homology arms (Supplementary Fig. 8). We found that nucleofection and gRNA-1 from both protocols worked for both the H1 and H9 lines, but we moved forwards with the parental lines from Skarnes et al. because the bands for these cells appeared to be more pronounced. Following visualization of the correct band on a 1% agarose gel, the band was gel extracted (Zymogen, D4007) and Sanger sequenced to verify the correct insertion. We then thawed H1 and H9 parental cryovials and FACS sorted DAPI-negative single hPSCs into four 96-well tissue culture plates containing mTESR Plus and CloneR. Small to medium-sized colonies were visible in 108 of the 384 wells after 1 week, and clonal colonies were split into 24-well plates, expanded and split for genomic DNA isolation and cryostorage. Extensive PCR genotyping and sequencing using multiple primer pairs were used to identify two hPSC clones (clones 9 and 25 in H9) with the correct knock-in (Supplementary Fig. 8c).

We proceeded to directed differentiation of hPSCs into SMPCs with both knock-in clonal lines, and one line was able to produce a high yield of PAX7^+^ SMPCs based on FACS enrichment of ERBB3^+^NGFR^+^ cells, real-time PCR analysis and in vitro myotube fusion. Sorted SMPCs were expanded for 3 days in vitro after sorting and then approximately 1 million SMPCs were injected into 6-week-old mdx-D2-NSG Pax7-ablated mice that were pretreated with barium chloride. Two weeks following engraftment, mice were injected i.p. with AP1903 (10 μM in 100 μl saline) or control (0.1% DMSO in saline) every 2 weeks for 90 days (six total injections). Mouse human-transplanted tissues were then dissected and mounted on microscope slides, and we proceeded to same-day GeoMx spatial RNA-seq. A total of 59 ROIs were sequenced, including 18 regions containing PAX7^+^ cells, 23 regions containing hPSC myofibres and 18 regions containing human lamin AC^+^PAX7^–^ cells.

### GeoMx spatial RNA-seq

#### Slide preparation

Spatial RNA-seq was performed using human skeletal muscle tissues (limb) or mouse tissues (TA) engrafted with human cells. We determined that freshly sectioned muscle tissues yielded the best RNA quality; thus, tissues were flash-frozen in isopentane-chilled liquid nitrogen and sectioned at 10 µm for same-day RNA-seq analysis. To identify engrafted regions, TA muscle sections were collected every 500 µm on Superfrost Plus slides for tissue adherence, which allowed us to avoid the suggested baking steps at 65 °C (Nanostring) to further improve RNA quality. Muscle sections were first washed with DPBS and fixed with 4% PFA for 30 min. To expose RNA targets, we empirically determined that tissues needed to be treated with 0.025 µg ml^–1^ proteinase K for 5 min at 37 °C as higher concentrations cleaved antigen binding sites. For in situ hybridization, human whole-transcriptome RNA probes (Nanostring) were applied to the tissues, which were then covered with a Grace Bio-Labs HybriSlip and incubated in a hybridization chamber at 37 °C overnight. The following day, the HybriSlips were removed in 2× SSC-T buffer and tissue slides were washed twice for 25 min with a solution composed of 4× SSC and deionized formamide. Washing and staining of slides were performed using Grace Bio-Labs silicon coverslips to avoid hydrophobic pen usage. To add morphology markers, we blocked with buffer W for 30 min and then added primary antibodies, including antibodies to PAX7 (DSHB; 1:30), spectrin (1:80), lamin AC (ThermoFisher; 1:80) and ACTC1 (Sigma; 1:150), diluted in buffer W, for 90 min. Tissues were washed three times with DPBS, and secondary antibodies conjugated to Alexa Fluor 532, 594 and 647 were added (at a 1:250 dilution) alongside Syto13 nuclear dye (Nanostring; 1:15) in DPBS for 60 min. Tissues were washed in DPBS and taken to the GeoMx machine for scanning and selection of ROIs.

#### RNA probe collection

Selection of ROIs and segmentation were based on fluorescence intensity for PAX7^+^ cells (PAX7^+^lamin AC^+^), myofibres (spectrin^+^lamin AC^+^ACTC1^±^) and non-myogenic human cells (lamin AC^+^, negative for other markers). The GeoMx platform photocleaves and collects RNA probes within segmented ROIs for sequencing. A list of all 125 ROIs collected for sequencing is shown in Supplementary Fig. 9b. We sequenced at twice the recommended coverage (200 reads per µm^2^) for a total read count of 161 million reads.

#### Analysis

FASTQ files were aligned to a human reference genome (hg38), and data were uploaded to the GeoMx platform to begin analysis. We first ran a general quality control, and all ROIs passed the quality control analysis. To filter the data, we filtered at 1% of the limit of quantification and higher than the user-defined value of 2 and normalized using the quartile-3 (Q3) method. To compare different cell types or treatments to generate volcano plot data, linear mixed model (LMM) statistical tests with Benjamini–Hochberg correction were performed. The .csv files from GeoMx analysis served as the input for generating volcano plots in R Studio using the EnhancedVolcano program. PCA and UMAP plots were generated using modified custom scripts on the GeoMx platform.

### Statistics and reproducibility

Please see individual Methods sections for sample sizes and how statistical analysis was performed for each experiment. Unless noted otherwise, all statistical *t* tests used were parametric, unpaired and two tailed. All one-way ANOVA analyses were corrected for multiple comparisons and assumed a Gaussian distribution. GraphPad Prism version 9.3.1 was used to generate graphs and statistics. In general, randomization and blinding were performed where possible. For imaging of PAX7, regions were first blindly selected in the lamin AC fluorescence channel and PAX7 cell images were then captured without predetermining PAX7 locations. All image data were quantified by two or more users, and individual cells such as PAX7^+^ cells were marked on the original image so that multiple users could verify niche occupancy and PAX7 positivity. For GeoMx spatial RNA-seq, users were blinded to engraftment efficiency until images were scanned through the platform. To increase reproducibility, four hPSC lines were used in this study, including H9, H9 with a PAX7-GFP reporter, H9 with a luciferase reporter, H9 with an iCas9 gene, CDMD 1002 and CDMD 1006-1, and WTC-11. We also used two separate dystrophic mouse models (mdx-NSG and mdx-DBA/2-NSG) to confirm the presence of the human-only myofibres. Litter- and age-matched mice were selected at random for engraftment and re-injury experiments, and analyses between groups such as foetal versus adult engraftment were performed with blinding. No animals or data points were excluded from the study. No statistical method was used to predetermine sample size, but sample sizes for engraftment are similar to those reported in previous publications^14^. In general, data distribution was assumed to be normal, but this was not formally tested. The one set of experiments where data distribution was not assumed to be normal was in the engraftment distribution of SMPCs into mouse skeletal muscle. For these experiments, we sectioned multiple regions throughout the muscle and report engraftment efficiency across the entire length of the muscle (Fig. 1a and Extended Data Fig. 3b) or we report engraftment efficiency as the maximum number of engrafted myofibres from the cross-sectional region with the greatest engraftment, and these regions were used for statistical comparison (Extended Data Fig. 7a). For spatial RNA-seq data, all ROIs were validated by the user in addition to the software algorithms provided by the GeoMx DSP segment. If pixel intensities led to inaccurate segmentation, these data were excluded.

### Reporting summary

Further information on research design is available in the Nature Portfolio Reporting Summary linked to this article.

## Online content

Any methods, additional references, Nature Portfolio reporting summaries, source data, extended data, supplementary information, acknowledgements, peer review information; details of author contributions and competing interests; and statements of data and code availability are available at 10.1038/s41556-023-01271-0.

### Supplementary information

Reporting Summary

Supplementary Video 1 Video highlighting extensive hPSC SMPC engraftment after 30 days in mdx-NSG mouse muscle. Shown are human myofibres (dystrophin, green), human nuclei (lamin AC, red) and mouse myofibres (laminin, grey).

Supplementary Table 1 snRNA-seq data supporting Fig. 4b. **Tab 1**. UMAP population labels and percentages identified in mdx-NSG muscle with or without injury or Pax7 ablation. **Tab 2**. Differential gene expression of individual populations compared with all populations using datasets from combined mouse models.

Supplementary Table 2 Differential gene expression from snRNA-seq supporting Fig. 4e. **Tab 1**. Shown are *P* values, log_2_ fold change (FC), and expression (pct.1) of IIB.3 myofibres (Actc1^+^Myh4^+^) compared with all other cell populations (pct.2). Data are separated by mouse models; from left to right: mdx-NSG Pax7-DTX 8 days post injury, mdx-NSG Pax7-DTX no injury, mdx-NSG, and mdx-NSG 8 days post injury.

Supplementary Table 3 **Tab 1**. Meta-analysis datasheet containing all spatial RNA-seq information for ROIs (*N* = 122) including population identity formatted for upload into Nanostring GeoMx for further analysis. **Tabs 2–4**. Further output information about target, bioprobes and dataset summary generated from GeoMx. **Tabs 5–7**. RNA counts used to generate Figs. 6 and 7, and Extended Data Fig. 10. As RNA counts are normalized on the basis of sample input, datasets containing foetal engraftment, hPSC engraftment or all data combined produce slightly different RNA counts and gene cutoffs. Thus, separate tabs are required for accurate analysis.

Supplementary Table 4 Differential gene expression used to generate volcano plot in Fig. 6c. **Tabs 1–3** show genes upregulated in ACTC1^+^ and ACTC1^−^ foetal myofibres, *P* *<* 0.05.

Supplementary Table 5 Differential gene expression used to generate volcano plot in Fig. 6d. **Tabs 1–3** show genes upregulated in specific foetal PAX7 SMCs after injury or 4 months without an injury, *P* *<* 0.05.

Supplementary Table 6 Differential gene expression used to generate volcano plot in Fig. 7d. **Tabs 1–3** show upregulated genes in iCasp9 hPSC SMPC with our without AP1903, *P* *<* 0.05.

Supplementary Table 7 Differential gene expression used to generate volcano plot in Extended Data Fig. 9. **Tabs 1–3** show upregulated genes in PAX7^+^ hPSC SMPCs compared with PAX7^−^ hPSC SMPCs, *P* *<* 0.05. Data exclude myofibres.

Supplementary Table 8 Differential gene expression used to generate volcano plot in Fig. 7d. **Tabs 1–3** show genes upregulated in PAX7^+^ hPSC SMPCs compared with hPSC myofibres, *P* *<* 0.05.

### Source data

Source Data Fig. 1 Full sequence of iCaspase9 vector used for knock-in to 3′ untranslated region of ACTC1 in human embryonic stem cells for upload into Snapgene.

Source Data Fig. 2 Full sequence of gRNAs 1–3 used for knock-in to 3′ untranslated region of ACTC1 in human embryonic stem cells for upload into Snapgene.

Source Data Fig. 3 Unprocessed gel used for identifying knock-in conditions for iCaspase9.

Source Data Fig. 4 Unprocessed gel used for identifying single-cell H9 (clone 9).

Source Data Fig. 5 Unprocessed gel used for identifying single-cell H9 (clone 25).

Source Data Fig. 6 Unprocessed gel used for gel extraction and sequencing from H9 clones 9 and 25.

## Data Availability

Single-nucleus RNA-seq data that support the findings of the studies in *Pax7*-knockout mice have been deposited in the Gene Expression Omnibus (GEO) under accession code GSE241368. Spatial RNA-seq code data that support the findings of the engraftment studies have been deposited in GEO under accession code GSE243875. Source data are provided with this paper. All other data supporting the findings of this study are available from the corresponding author on reasonable request. Source data are provided with this paper.

## Code availability

Spatial RNA-seq code data that support the findings of the engraftment studies have been deposited in GitHub at https://github.com/mhickslab/Spatial_RNA_Seq_Myogenic_Engraftment and can be accessed for further analysis in GeoMx software or in R progamming software.
