# Publisher Correction: Regenerating human skeletal muscle forms an emerging niche in vivo to support PAX7 cells

**DOI:** 10.1038/s41556-023-01320-8

**Published:** 2023-12-18

**Authors:** Michael R. Hicks, Kholoud K. Saleh, Ben Clock, Devin E. Gibbs, Mandee Yang, Shahab Younesi, Lily Gane, Victor Gutierrez-Garcia, Haibin Xi, April D. Pyle

**Affiliations:** 1 Eli and Edythe Broad Center of Regenerative Medicine and Stem Cell Research, University of California, Los Angeles, CA USA; 2 Department of Microbiology, Immunology, and Molecular Genetics, University of California, Los Angeles, CA USA; 3 Physiology and Biophysics, University of California, Irvine, CA USA; 4 Molecular, Cellular & Integrative Physiology Program, University of California, Los Angeles, CA USA; 5 Molecular Biology Institute, University of California, Los Angeles, CA USA; 6 CIRM Bridges Program, California State University, Northridge, CA USA; 7 Jonnson Comprehensive Cancer Center, University of California, Los Angeles, CA USA

**Keywords:** Muscle stem cells, Regeneration, Musculoskeletal development

Correction to: *Nature Cell Biology* 10.1038/s41556-023-01271-0, published online 2 November 2023.

In the version of the article initially published, the Methods were missing and are now included in the HTML and PDF version of the article.

