# Supplementary material for: Regenerating human skeletal muscle forms an emerging niche in vivo to support PAX7 cells
Source: Nat Cell Biol. 2023 Nov 2;25(12):1758–73. doi: 10.1038/s41556-023-01271-0 (PMC10709143; doi:10.1038/s41556-023-01271-0)
Supplement: Supplementary file 1 — Reporting Summary [file 41556_2023_1271_MOESM1_ESM.pdf]

nature portfolio

Corresponding author(s): Hicks MR and Pyle AD

Last updated by author(s): 9.22.23

Reporting Summary

Nature Portfolio wishes to improve the reproducibility of the work that we publish. This form provides structure for consistency and transparency in reporting. For further information on Nature Portfolio policies, see our Editorial Policies and the Editorial Policy Checklist.

Statistics

For all statistical analyses, confirm that the following items are present in the figure legend, table legend, main text, or Methods section.

n/a	Confirmed	
☐	☒	The exact sample size (n) for each experimental group/condition, given as a discrete number and unit of measurement
☐	☒	A statement on whether measurements were taken from distinct samples or whether the same sample was measured repeatedly
☐	☒	The statistical test(s) used AND whether they are one- or two-sided<br>*Only common tests should be described solely by name; describe more complex techniques in the Methods section.*
☐	☒	A description of all covariates tested
☐	☒	A description of any assumptions or corrections, such as tests of normality and adjustment for multiple comparisons
☐	☒	A full description of the statistical parameters including central tendency (e.g. means) or other basic estimates (e.g. regression coefficient) AND variation (e.g. standard deviation) or associated estimates of uncertainty (e.g. confidence intervals)
☐	☒	For null hypothesis testing, the test statistic (e.g. F, t, r) with confidence intervals, effect sizes, degrees of freedom and P value noted<br>*Give P values as exact values whenever suitable.*
☒	☐	For Bayesian analysis, information on the choice of priors and Markov chain Monte Carlo settings
☐	☒	For hierarchical and complex designs, identification of the appropriate level for tests and full reporting of outcomes
☒	☐	Estimates of effect sizes (e.g. Cohen's d, Pearson's r), indicating how they were calculated

Our web collection on statistics for biologists contains articles on many of the points above.

Software and code

Policy information about availability of computer code

Data collection	Data collection was performed using the following imaging platforms: Zeiss Axio Observer 1, a Nikon Eclipse 600, a Zeiss LSM 880 confocal microscope, and an IVIS in vivo imager. We used the NovoSeq 6000 sequencer for both bulk RNA-Seq and single nuc RNA seq. The BD FACS ARIA II was used for all flow cytometry experiments.
Data analysis	We used GraphPad PRISM 9.3.1 to perform statistical comparisons, which included one-way ANOVA for multiple sample comparisons, P<0.05 and t-test for comparison of two groups, P<0.05. FlowJo was used for FACS analyses and determining population percentages within gates. Zeiss Zen Blue software, Nikon Elements, and Imaris by Bitplane were used for image quantification. For bulk RNA-SEQ samples were aligned in HiSAT2 to the human genome (hg19, UCSC) and Ensembl Homo Sapiens GTF file (GRCh37). String Tie and DESEQ were used to evaluate differential gene expression using a false discovery rate of 0.01. For single nucleus RNA Sequencing, data were aligned using Cell Ranger by 10X genomics to the human genome (hg19, UCSC). R studio packages Seurat and CellChat were used for analysis. NCBI DAVID was used for functional annotation of differential gene expression. For additional information, please see extended experimental procedures.

For manuscripts utilizing custom algorithms or software that are central to the research but not yet described in published literature, software must be made available to editors and reviewers. We strongly encourage code deposition in a community repository (e.g. GitHub). See the Nature Portfolio guidelines for submitting code & software for further information.

Data

Policy information about availability of data

All manuscripts must include a data availability statement. This statement should provide the following information, where applicable:

- Accession codes, unique identifiers, or web links for publicly available datasets
- A description of any restrictions on data availability
- For clinical datasets or third party data, please ensure that the statement adheres to our policy

All data is available upon request. All data will be uploaded to NIH NCBI Geo and https://aprilpylelab.com

Field-specific reporting

Please select the one below that is the best fit for your research. If you are not sure, read the appropriate sections before making your selection.

☒ Life sciences ☐ Behavioural & social sciences ☐ Ecological, evolutionary & environmental sciences

For a reference copy of the document with all sections, see nature.com/documents/nr-reporting-summary-flat.pdf

Life sciences study design

All studies must disclose on these points even when the disclosure is negative.

Sample size	Sample sizes were chosen based on pilot studies of the experiment being performed. Please see supplemental methods. Across cell lines, we engrafted mice with ERBB3+NGFR+ sorted hPSC-SMPCs N=7 for figure 1. For the re-injury engraftment studies, engraftment was performed in N=6-14 mice. For bulk RNA-seq a total of 14 samples were evaluated (N=3 fetal week 10, N=3 fetal week 18, N=3 adult SC, N=5 hPSC SMPC). The injury response in Pax7 knockout mice with or without injury was performed on N=5 mice. We then performed engraftment studies in Pax7 knockout using N=12 mice for human fetal and N=4 mice for human adult satellite cells.
Data exclusions	Some Pax7 knockout mdx-NSG mice died from dehydration as a result of tamoxifen induced diphtheria toxin. These mice were excluded from the studies. For human PAX7 counts (Figure 1) we included images containing at least 1 PAX7+ cells; however, in the supplement (Figure S2) we further quantified all engrafted regions containing human cells regardless of PAX7 numbers.
Replication	To determine if human only myofibers was observed in multiple mouse models, we included both mdx-NSG and a more severe dystrophic mouse model mdx-D2-NSG mice. Throughout the study, we performed FACS enrichment and engraftment studies on over 20 biological specimens from fetal and adult. All attempts at replication were successful.
Randomization	Mice within a litter ages 6-8 weeks, were randomly selected for engraftment studies. For PAX7 cells counts, we randomly selected regions with high numbers of human cells (marked by Lamin AC) and then imaged that region for PAX7. Treatment groups were processed identically throughout all experiments so that each group had equal probability of responding to treatment and to limit bias of results. For transplantation studies equal numbers of male and female mdx-NSG mice were used at random. All animals were housed in the same area and no preference was given to which mouse received a given treatment/transplantation.
Blinding	Depending on the experiment, analyses were performed in a blinded fashion. Microscope slides labels were covered with tape and recorded as i.e. A, B, C, etc. Scoring of all image quantification data was recorded so that multiple users could reevaluate data and derive the same results. Flow cytometry and sequencing was performed by core personal who were blinded to the samples, labeled as AP1, AP2, etc. For animal studies, sectioning and staining were blinded with animal numbers. After image quantification of h-Lamin AC and h-Dystrophin, key was used to identify groups. Investigators were blinded to analysis on image quantifications.

Reporting for specific materials, systems and methods

We require information from authors about some types of materials, experimental systems and methods used in many studies. Here, indicate whether each material, system or method listed is relevant to your study. If you are not sure if a list item applies to your research, read the appropriate section before selecting a response.

Materials & experimental systems

n/a	Involved in the study	
☐	☒	Antibodies
☐	☒	Eukaryotic cell lines
☒	☐	Palaeontology and archaeology
☐	☒	Animals and other organisms
☒	☐	Human research participants
☒	☐	Clinical data
☒	☐	Dual use research of concern

Methods

n/a	Involved in the study	
☒	☐	ChIP-seq
☐	☒	Flow cytometry
☒	☐	MRI-based neuroimaging

Antibodies

Antibodies used	All antibodies used in this study are commercially available and are described in the methods.<br>Spectrin: Leica NCL-SPEC1<br>Lamin A/C: Vector VP-L550<br>Laminin: Sigma L9-393<br>ACTC1: Sigma A9357<br>MYH3: DSHB BF-45<br>MYH4: DSHB BF-F3<br>M-Cadherin: BD Biosciences 61160<br>Dystrophin: DSHB MANDYS106
Validation	PCR primers were validated for efficiency using a 6 serial dilutions with 5-fold dilution curves. Only primers with >90% efficiency were used. Antibodies were validated using human or mouse tissue positive controls and isotype negative controls. Human PAX7 cells required the colocalization of 3 markers (Lamin AC, PAX7, and DAPI) to be scored. All antibodies were validated using isotype controls and based on previous publications (see Hicks et al NCB 2018). Serial dilutions were used to identify signal to noise ratio.

Eukaryotic cell lines

Policy information about cell lines

Cell line source(s)	This study used primary cells and hPSCs. Adult human cells were obtained from tissue donations by NIH NDRI. Fetal human cells were provided by ABR. Cell lines used included hESC line: H9 (Wicell) and UCLA stem cell core, a hPSC PAX7 reporter line (generated in the Pyle lab), and a hPSC bioluminescent reporter line (generated in Pyle Lab).
Authentication	All hPSC lines were authenticated by karyotype analyses and teratoma formation.
Mycoplasma contamination	Yes, all cell lines were tested and were mycoplasma free.
Commonly misidentified lines<br>(See ICLAC register)	No commonly misidentified cell lines were used.

Animals and other organisms

Policy information about studies involving animals; ARRIVE guidelines recommended for reporting animal research

Laboratory animals	Animals used were both male and female mice aged 6-8 weeks. We used mdx-NSG mice and mdx-D2-NSG mice generated in our laboratory. These mice were backcrossed for several >5 generations to original mdx background while retaining Scid and IL2 knockout. C57BL6J (C57)-NSG, Mdx-NSG, and Mdx-DBA/2-NSG were crossed to Rosa-DTA mice (Jax #009669) or Pax7-Cre/ERT2 (Jax #017763) mice. Both Rosa-DTA and Pax7-Cre/ERT2 were crossed to C57-NSG, Mdx-NSG, and Mdx-DBA/2-NSG strains for 3-5 generations prior to the start of Pax7 ablation experiments. Congenic homozygous Pax7-Cre/ERT2 males were then crossed to homozygous Rosa-DTA mice females, and heterozygous F1 Pax7 Cre/WT Rosa DTA/WT mice were used for Pax7 ablation studies. All animals were kept in sterile housing maintained by the UCLA or UC IRVINE vet staff in A+ barrier facilities. They were given standard 12 hr dark/light cycles.
Wild animals	No wild animals were used in this study.
Field-collected samples	No samples were collected in the field.
Ethics oversight	All animal work was approved by the UCLA ARC 2006-119.

Note that full information on the approval of the study protocol must also be provided in the manuscript.

Flow Cytometry

Plots

Confirm that:

☒ The axis labels state the marker and fluorochrome used (e.g. CD4-FITC).

☒ The axis scales are clearly visible. Include numbers along axes only for bottom left plot of group (a 'group' is an analysis of identical markers).

☒ All plots are contour plots with outliers or pseudocolor plots.

☒ A numerical value for number of cells or percentage (with statistics) is provided.

Methodology

Sample preparation	Lin-ERBB3+NGFR+fetal weeks 9-20 or Lin-CD82+NCAM+ adult SCs years 30-60 were enriched from primary tissues

Sample preparation	using FACS and immediately transplanted into mice models. Primary SMPC/SCs were dissociated as described (Hicks et al., 2018), washed in HBSS, and concentrated at 1x104-1x106 cells/per 5µls HBSS.
Instrument	BD FACS ARIA II
Software	DIVA
Cell population abundance	For adult CD82+CD56+ satellite cells represented 5-25% of the lineage negative (CD45-CD235-PDGFRa-CD31-) cell fraction and 0.5-2% of the total population. For fetal week 17-18 ERBB3+NGFR+ progenitor cells represented 15-35% of the lineage negative (CD45-CD235-PDGFRa-CD31-) cell fraction and 2-6% of the total population. For hPSC SMPC ERBB3+NGFR+ progenitor cells represented 10-30% of the lineage negative (HNK1-) cell fraction and 5-25% of the total population.
Gating strategy	Cells were gated on FSC vs SSC for size and complexity; then sorted on FSC-H vs. FSC-A for singlet cells. Cells were then gated on FSC vs live/dead viability dye. We then combined lineage negative antibodies into a dump channel. Lineage negative muscle stem or progenitor cells were then sorted for double positive cell populations. Fluorescent minus one (FMO) gates were used for all FACS experiments as gating controls.

☒ Tick this box to confirm that a figure exemplifying the gating strategy is provided in the Supplementary Information.
