# Supplementary figures and images for: Regenerating human skeletal muscle forms an emerging niche in vivo to support PAX7 cells
Source: Nat Cell Biol. 2023 Nov 2;25(12):1758–73. doi: 10.1038/s41556-023-01271-0 (PMC10709143; doi:10.1038/s41556-023-01271-0)

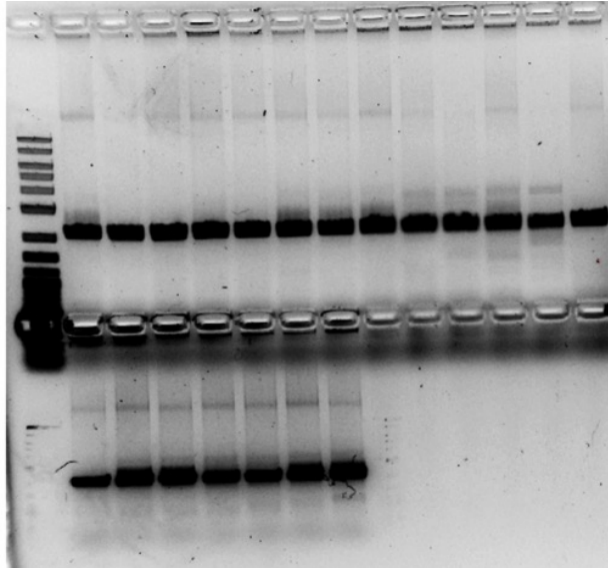

Supplement: Supplementary file 13 — Unprocessed gel used for identifying knock-in conditions for iCaspase9. [file 41556_2023_1271_MOESM13_ESM.pdf]

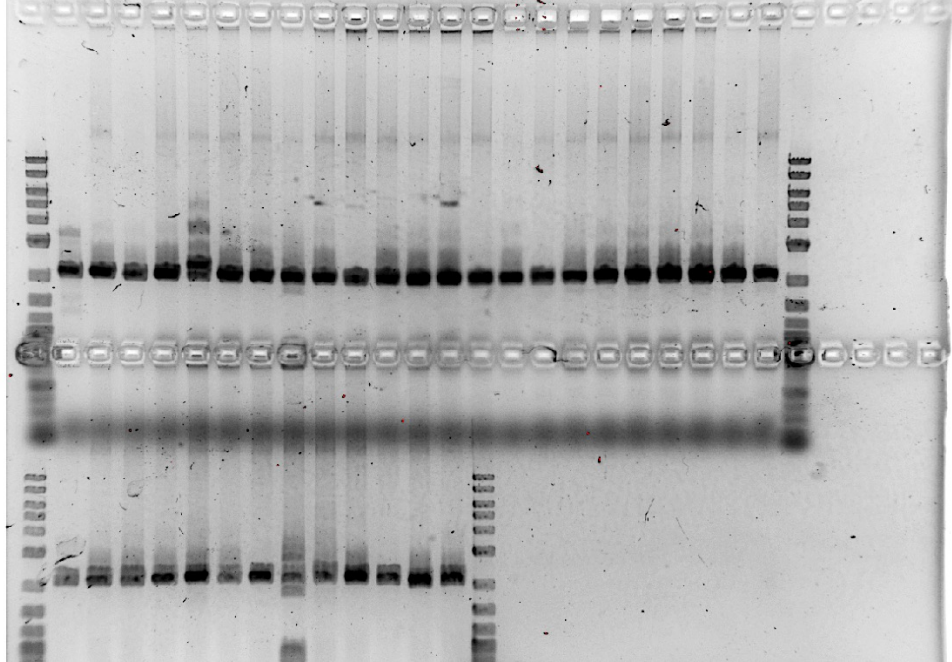

Supplement: Supplementary file 14 — Unprocessed gel used for identifying single-cell H9 (clone 9). [file 41556_2023_1271_MOESM14_ESM.pdf]

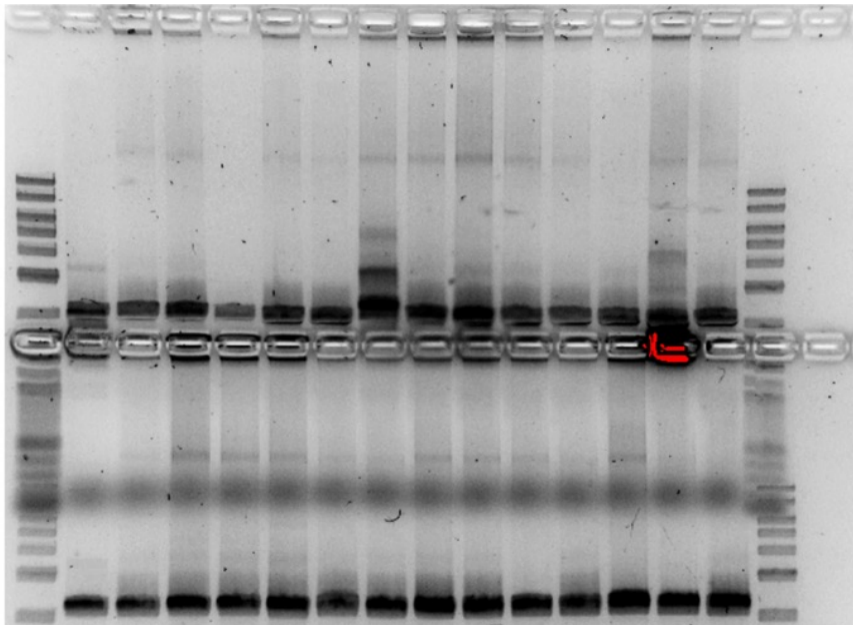

Supplement: Supplementary file 15 — Unprocessed gel used for identifying single-cell H9 (clone 25). [file 41556_2023_1271_MOESM15_ESM.pdf]

Next attachment

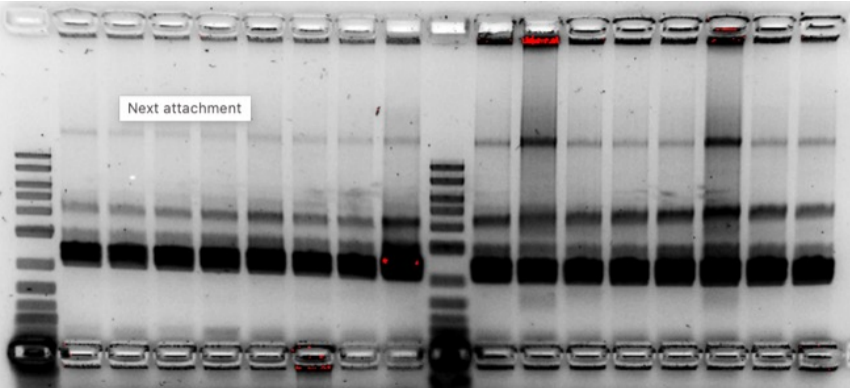

Supplement: Supplementary file 16 — Unprocessed gel used for gel extraction and sequencing from H9 clones 9 and 25. [file 41556_2023_1271_MOESM16_ESM.pdf]
